# Multifunctional Hydrogel Flakes: An Innovative Approach to Localized Delivery of Temozolomide

**DOI:** 10.1021/acsami.5c13013

**Published:** 2025-09-09

**Authors:** Aleksandra Krajcer, Alicja Hinz, Monika Bzowska, Adrian Grzonka, Sylwia Stankiewicz, Kamil Kornaus, Bartosz Trzewik, Kinga Wójcik, Ewelina Grzywna, Joanna Lewandowska-Łańcucka

**Affiliations:** † Doctoral School of Exact and Natural Sciences, 37799 Jagiellonian University, Prof. St. Łojasiewicza 11, Kraków 30-348, Poland; ‡ Faculty of Chemistry, Jagiellonian University, Gronostajowa 2, Kraków 30-387, Poland; § Department of Cell Biochemistry, Faculty of Biochemistry, Biophysics and Biotechnology, Jagiellonian University, Gronostajowa 7, Kraków 30-387, Poland; ∥ Faculty of Materials Science and Ceramics, 49811 AGH University of Krakow, Mickiewicza 30, Kraków 30-059, Poland; ⊥ Faculty of Biochemistry, Biophysics and Biotechnology, Jagiellonian University, Gronostajowa 7, Kraków 30-387, Poland; # Department of Neurosurgery and Neurotraumatology, 49573 Jagiellonian University Medical College, Św. Anny 12, Kraków 31-008, Poland

**Keywords:** glioblastoma, temozolomide, hydrogels, vancomycin, hemostatic properties, local therapy

## Abstract

The multifunctional systems presented here introduce an innovative and deeply thought-out approach to the more effective and safer use of temozolomide (TMZ) in treating glioma. The developed hydrogel-based flakes were designed to address the issues of local GBL therapy, bacterial neuroinfections, and the bleeding control needed during tumor resection. The materials obtained comprise TMZ and vancomycin (VANC) loaded into cyclodextrin/polymeric capsules and embedded into gelatin/hyaluronic acid/chitosan-based hydrogel films cross-linked with genipin. The calcium cations were also incorporated into the systems to facilitate the hemostatic property. That designed formulation was then freeze-dried to serve in flake-like forms, enabling the lining of the surgery site during surgical resection, transferring both TMZ and VANC directly into the surrounding brain parenchyma (the most common site of recurrence). Such a route of drug delivery represents unique features, since systemic side effects are minimized while the drug dose is increased. The developed systems were characterized by their designed functionalities. Therefore, biological evaluation *in vitro/ex vivo*, antibacterial activity tests *in vitro*, drug release studies, and physicochemical feature assessments were performed. Our results confirmed that the presented multifunctional long-acting delivery system allows for sustained TMZ release and possesses antimicrobial properties, while simultaneously displaying the hemostatic potential. Overall, we analyzed the unique clinical conditions related to the local CNS drug administration, providing each with dedicated solutions. The presented methods and results describe the invention and its interactions with the environment, an essential step toward clinical application.

## Graphical abstract

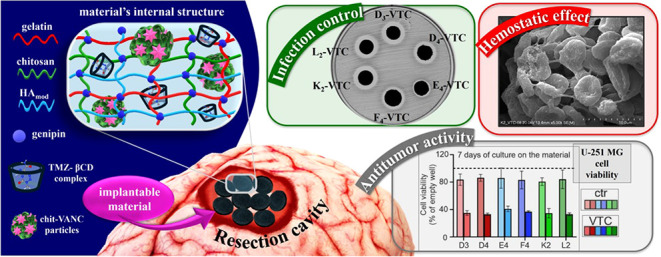

## 1 Introduction

Glioblastoma (GBL) is the most common malignant central nervous system (CNS) tumor in adults, which represents a significant challenge for neurooncology.^1,2^ The clinically approved treatment strategies involve surgical removal followed by systemic chemotherapy and radiotherapy.^3^ Despite this multimodal approach, the median survival of GBL patients is unfortunately still limited to 16–19 months.^4^ Temozolomide (TMZ) is currently the first-line chemotherapy for GBL.^4^ However, only a portion of the drug dose crosses the blood–brain barrier (BBB) since TMZ undergoes chemical degradation under physiological conditions, resulting in the production of the active metabolite 5-(3-methyl-triazen-1-yl)­imidazole-4-carboxamide (MTIC), which is unable to penetrate the brain parenchyma.^5^ Thus, the TMZ’s hydrolysis before reaching the central nervous system (CNS) significantly reduces its therapeutic efficacy and necessitates the use of multiple high doses, which expose the patient to many systemic adverse effects.^6^ Therefore, a promising strategy for GBL therapy is to combine TMZ with devices/vehicles designed for local administration that provide its transportation straight to the brain, overcoming the BBB.^7^ Moreover, the further benefits of local delivery lie in the potential to increase the drug bioavailability and considerably reduce the toxicity observed with systemically administered chemotherapy.^8^

To date, the only clinically approved implantable therapeutic agent for the treatment of GBL is Gliadel, a material based on biodegradable polymer wafers of poly­[bis­(p-carboxyphenoxy propane) sebacic acid] and loaded with the anticancer drug carmustine.^1,9^ Gliadel has increased overall survival in patients with recurrent GBL and newly diagnosed GBL.^10^ However, its use in the clinic remains controversial because of its unproven efficacy.^11^ Additionally, the stiff matrix of Gliadel wafers is a significant cause of adverse events, such as seizures, edema, meningitis, and increased risk of wound infections, occurring in patients undergoing implantation.^12^ Furthermore, it was reported that Gliadel suffers from the “sink effect”—the drug is being released from the wafers and washed away into systemic circulation due to excessive diffusion of the drug.^9^ Therefore, to overcome these limitations, a novel approach based on the TMZ carriers in the form of β-cyclodextrin and their use in conjunction with hydrogel-based films is proposed within this work.

The biopolymeric (gelatin/hyaluronic acid/chitosan) hydrogel-based films serve herein as a system for the local delivery of active substances, ensuring their localization/adhesion at the implantation site while maintaining their structure and biological properties. Gelatin (Gel) is a biocompatible and biodegradable water-soluble biopolymer obtained from collagen by hydrolytic degradation. Thus, it may serve as a cost-effective alternative to this compound. Gelatin’s particularly attractive biological feature is the presence of the primary structure “RGD” (arginine-glycine-aspartic acid sequence), which supports cell adhesion and proliferation.^13^ Moreover, Gel is the main component of a hemostatic neurosurgeon device (Clinisponge).^14^ Chitosan (Chit) and hyaluronic acid (HA) are linear polysaccharides widely applied in gene therapy, tissue engineering, and drug delivery, also for some CNS-related disorders, since they possess many unique advantages such as biodegradability, biocompatibility, and mucoadhesivity.^15,16^ Importantly, HA is a highly efficient targeting molecule for cancer therapy that can bind to the HA receptors (transmembrane glycoprotein CD44), overexpressed in many cancer cells, including malignant glioma.^17^ Hydrogels of properly selected chemical composition allow the preparation of materials characterized by biocompatibility and increased structural integrity.^18^ To fabricate the hydrogels with sufficient mechanical stability and a prolonged degradation profile, chemical cross-linking achieved by the formation of strong covalent bonds between the functional polymeric groups and genipin was applied to this work. Genipin is a naturally occurring cross-linking agent of low cytotoxicity and has the ability to efficiently cross-link biomacromolecules with primary amino groups.^19^ Furthermore, it was also reported that genipin may serve as a therapeutic agent since it exhibits anti-inflammatory and neuroprotective properties.^20^

Regarding implantation, it is essential to mention that associated neuroinfections constitute a significant concern for healthcare systems and, therefore, need to be considered when designing local brain delivery systems.^21^ Vancomycin (VANC) is a broad-spectrum glycopeptide antibiotic recommended for treating brain abscesses in postoperative neurosurgical patients.^22^ The effective treatment requires long-term (usually 4–8 weeks) hospitalization, which is associated with high costs and inconvenience for patients. Furthermore, the long-term intravenous administration of VANC may cause serious side effects.^23^ Therefore, direct local antibiotic delivery via biodegradable devices is highly desired in treating postoperative neuroinfections since both the toxicity associated with antibiotic treatment and the expenses involved with long-term hospitalization are reduced, while the local therapeutic concentration is achieved.^24,25^ The system fabricated in this study also addresses this issue. To provide antibacterial features to the developed materials, vancomycin was first encapsulated in chitosan-based nanocapsules and then added to polymeric films. Herein, genipin cross-linked hydrogels with TMZ/VANC-loaded cyclodextrin/polymeric carriers embedded were freeze-dried to serve in solid form. The designed flakes will be delivered to the selected brain area during surgical resection, transferring TMZ and VANC directly into the surrounding brain parenchyma.

Finally, given the delivery route (during tumor resection), bleeding control must also be considered. Recent reports present the development of injectable hemostatic hydrogels using proteins such as gelatin, collagen, and polysaccharide-based polymers such as chitosan and hyaluronic acid.^26,27^ However, the chitosan-based hydrogel gained the greatest interest as a hemostatic agent. Commercially available, FDA-approved Chit-based dressings are HemCon, Celox Gauze, Celox Rapid, ChitoGauze, Chitoflex, TraumaStat, and Celox.^28^ The mechanism of action is that the cationic amine groups of Chit interact with the anionic membrane of red blood cells (RBCs) to seal the bleeding site. Chitosan has also been reported to cause the activation and aggregation of platelets (PLTs). Various strategies have been utilized to improve the hemostatic potential of chitosan-based hydrogels, including inorganic component addition. Calcium ions play a crucial role in the coagulation cascade by facilitating the assembly of coagulation complexes and enabling fibrin stabilization via the activation of factor XIII.^29^ Therefore, supplementation with calcium chloride was additionally utilized to enhance/support the hemostatic potential of the prepared systems so that the resulting hybrids would aid in RBC/PLT aggregation and simultaneously cause the activation of factors involved in the coagulation cascade.

One can assume that, developed within this study, the novel multifunctional long-acting delivery systems will allow for sustained TMZ release, improving the effect of therapy, and possess antimicrobial features and hemostatic potential while being administered directly into brain parenchyma. We have focused on the physicochemical and biological *in vitro/ex vivo* evaluation of the designed hybrids to provide evidence for their potential in GBL therapy. To our knowledge, the materials for such multifeatures have not yet been presented in the literature.

## 2 Materials and Methods

### 2.1 Materials

Gelatin from bovine skin (Type B, ∼225 g Bloom, Sigma-Aldrich), chitosan (5–20 mPa·s, 0.5% in 0.5% acetic acid at 20 °C, deacetylation value, min. 70.0%, TCI), lysine-modified hyaluronic acid (HA_mod_) obtained by functionalization of hyaluronic acid (Mw ∼ 1.5–1.8 × 10^6^ Da, Sigma-Aldrich) using the procedure described by us earlier^30^ (a substitution degree of about 18% was calculated based on ^1^H NMR spectroscopy), genipin (Challenge Bioproducts Co., 98%), vancomycin hydrochloride (pharmaceutical secondary standard, Sigma-Aldrich), sodium tripolyphosphate (≥98%, Sigma-Aldrich), temozolomide (certified reference material, pharmaceutical secondary standard, Sigma-Aldrich), beta-cyclodextrin (≥97%, Sigma-Aldrich), 2,4,6-trinitrobenzenesulfonic acid (5.2%, Sigma-Aldrich), and calcein (suitable for fluorometric determination of Ca and suitable for EDTA titration of Ca in the presence of Mg, Sigma-Aldrich). Reoxcel, TachoSil, and Clinisponge were purchased from the authorized sellers.

### 2.2 Synthesis of Hydrogel-Based Flakes, Vancomycin-Loaded Chitosan Particles, TMZ-βCD Complexes, and Multifunctional Composites with All Those Components

#### 2.2.1 Hydrogel-Based Flakes

A set of biopolymeric solutions at a concentration of 30 mg/mL each was prepared by dissolving gelatin (Gel) in deionized water (DI) at 37 °C, chitosan (Chit) in 1% acetic acid, and lysine-modified hyaluronic acid (HA_mod_) in 10× PBS (the 10× PBS buffer solution contains 1.37 M sodium chloride, NaCl; 27 mM potassium chloride, KCl; 43 mM disodium hydrogen phosphate, Na_2_HPO_4_; and 14 mM potassium dihydrogen phosphate, KH_2_PO_4_; pH was adjusted to 7.4). A genipin stock solution (44.2 mM) was prepared by dissolving the cross-linking agent in 10× PBS buffer. The synthesis of hydrogel-based flakes was initiated by mixing appropriate amounts of biopolymer solutions at various Gel:Chit:HA_mod_ weight ratios (Figure 1A) with the cross-linking agent (see Table 1). Polymeric weight ratios were counted per 1 mL of the mixture, which included 750 μL of biopolymeric solutions, 100 μL of 1% acetic acid, and 150 μL of genipin solution/genipin stock solution with 10× PBS additive to receive a cross-linking agent final concentration of 2.65, 4.64, or 6.63 mM.

**Figure 1:**
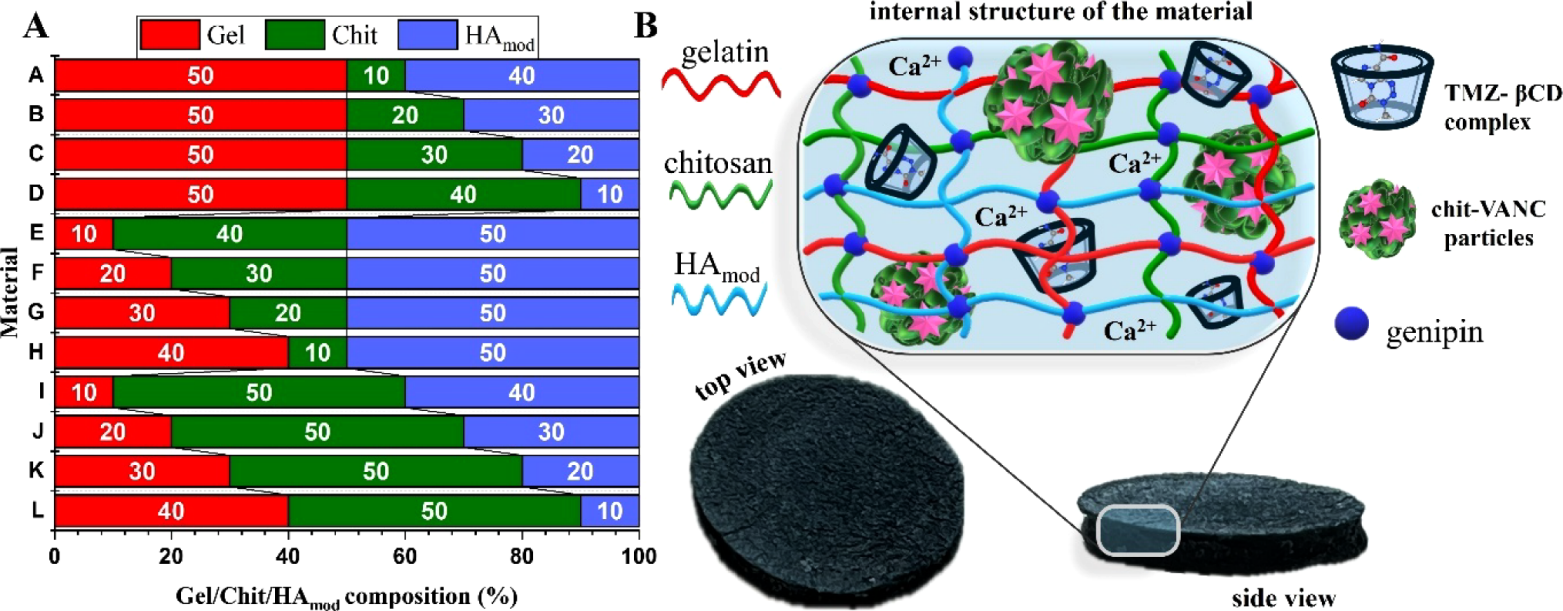
(A) Tested biopolymeric matrix composition depending on the weight ratios of individual biopolymers; (B) schematic illustration of the material, including its actual representation and internal structure.

**Table 1:** Biopolymeric Matrices Prepared Using Various Biopolymer Weight Ratios and Three Distinct Concentrations of the Crosslinking Agent

	Matrices of composition and cross-linking agent concentration		
Gel:Chit:HA_mod_ weight ratio	Genipin *c* = 2.65 mM	Genipin *c* = 4.64 mM	Genipin *c* = 6.63 mM
5/1/4	A2	A3	A4
5/2/3	B2^a^	B3	B4
5/3/2	C2^a^	C3^a^	C4
5/4/1	D2	D3	D4
1/4/5	E2	E3	E4
2/3/5	F2	F3	F4
3/2/5	G2	G3	G4
4/1/5	H2	H3	H4
1/5/4	I2	I3	I4
2/5/3	J2	J3	J4
3/5/2	K2	K3	K4
4/5/1	L2	L3	L4

a Materials that did not form an integrated biopolymeric network during synthesis.

Next, 8.56 mL of each sol with genipin (A2/A3/A4/B2/B3/B4/C2/C3/C4/D2/D3/D4/E2/E3/E4/F2/F3/F4/G2/G3/G4/H2/H3/H4/I2/I3/I4/J2/J3/J4/K2/K3/K4/L2/L3/L4) was poured into a round silicone mold with a 6 cm diameter, placed in a Petri dish, and incubated at 37 °C for 24 h to form a hydrogel. After gelation, the material was rinsed three times with DI, frozen, and lyophilized.

#### 2.2.2 Vancomycin-Loaded Chitosan Particles (Chit-VANC)

An ionic gelation with tripolyphosphate (TPP) was employed to obtain vancomycin-loaded chitosan particles. A chitosan solution (5 mg/mL in 1% acetic acid) was heated to 60 °C, filtered through a 0.45 μm syringe filter, and used as the solvent for dissolving 15 mg of vancomycin (VANC) in a total volume of 7 mL. The resulting solution was placed on a magnetic stirrer (300 rpm), and 5.55 mL of a cold TPP solution (1.6 mg/mL in water), filtered through a 0.22 μm syringe filter, was added. The mixture transitioned from transparent to opalescent and was stirred for 30 min to allow particle formation (chit-VANC).

#### 2.2.3 TMZ-βCD Complexes (T-βCD)

Host–guest complexes in the convention of beta-cyclodextrin (βCD)—temozolomide (TMZ) were formed based on the protocol described previously.^31^ Briefly, 3.5 g of βCD was suspended in 0.1 M HCl to obtain 20 g of the total mixture weight. The system was subjected to vigorous mixing for 15 min. In another vessel, 300 mg of TMZ was weighed, and the previously prepared suspension of βCD in 0.1 M HCl was poured into the vial. Then, the suspension was vortexed for 9 min. Subsequent addition of 0.1 M HCl to 40 mL was followed by one h of incubation at 37 °C with shaking (250 rpm). The TMZ-βCD complexes (T-βCD) were isolated after centrifugation (6000 rpm for 5 min) and freeze-drying.

#### 2.2.4 Multifunctional Composites in the Form of Lyophilized Flakes

Multifunctional composites are hydrogel-based flakes composed of biopolymeric matrices with a selected composition and three additives: vancomycin-loaded chitosan particles (Chit-VANC), TMZ-βCD inclusion complexes, and Ca^2+^ ions (Figure 1B).

The selected matrices included D3, D4, E4, F4, K2, and L2. Their synthesis followed a protocol analogous to that of hydrogel-based flakes, with slight modifications. Specifically, proper volumes of biopolymeric solutions (30 mg/mL Gel in DI, 30 mg/mL Chit in 1% CH_3_COOH, 30 mg/mL HA_mod_ in 10× PBS) were mixed to achieve Gel:Chit:HA_mod_ weight ratios corresponding to D3, D4, E4, F4, K2, and L2 (see Table 1). Then, the biopolymeric sols were mixed with various genipin volumes of stock solution (44.2 mM in 10× PBS) to receive a cross-linking agent final concentration of 2.65/4.64/6.63 mM. In this procedure, 100 μL of 1% CH_3_COOH per 1 mL of the sol was replaced with 100 μL of Chit-VANC dispersion. As a result, the final 1 mL of sol consisted of 750 μL of biopolymeric solutions, 100 μL of Chit-VANC particle formulation, and 150 μL of genipin stock solution, either alone or diluted in 10× PBS.

To ensure uniform sample preparation, each replicate was formed from 609 μL of the specific sol with particles, which were poured into the wells of a 24-well plate. The samples were incubated at 37 °C for 24 h, rinsed thrice with DI, and freeze-dried. Next, 1 mL of a 10 mg/mL CaCl_2_ solution containing 15 mg of TMZ-βCD complexes was added to each well with the flake-like materials. The samples were incubated at room temperature for 24 h with shaking (100 rpm). After incubation, the solution was withdrawn, and the materials were rinsed with DI and lyophilized to obtain D3-VTC, D4-VTC, E4-VTC, F4-VTC, K2-VTC, and L2-VTC composites.

### 2.3 Physicochemical Analysis of the Multifunctional System Components and the Prepared Composites

#### 2.3.1 Hydrogel-Based Matrices of Different Composition Characteristics

##### 2.3.1.1 Determination of −NH_2_ Groups in Biopolymeric Sols of Different Composition

To evaluate the number of amino groups available for cross-linking with genipin in sols of the tested Gel:Chit:HA_mod_ weight ratios, the widely described in the literature 2,4,6-trinitrobenzenesulfonic acid (TNBS) assay was employed.^32^ During the procedure, 0.4 mL of a specific sol (A/B/C/D/E/F/G/H/I/J/K/L) was mixed with 0.5 mL of 4% NaHCO_3_ (pH 8.5) and 0.5 mL of freshly prepared 0.05% TNBS. Then, the samples were incubated for 120 min at 40 °C. After that time, 1.6 mL of 6 M HCl was introduced to each vial, and the probes were left for 90 min at 60 °C. Subsequently, 2.5 mL of DI was added, and the samples were cooled to room temperature. Following this step, the systems were diluted 2-fold with DI, and their absorption spectra in the 200–650 nm range were registered. A quantitative analysis of −NH_2_ group content was performed based on a calibration curve for glycine solutions of known concentrations, comparing the absorption maximum at 340 nm.

##### 2.3.1.2 Swelling Evaluation

Biopolymeric genipin-cross-linked flakes of different types (A2/A3/A4/B3/B4/C4/D2/D3/D4/E2/E3/E4/F2/F3/F4/G2/G3/G4/H2/H3/H4/I2/I3/I4/J2/J3/J4/K2/K3/K4/L2/L3/L4) with 1 cm of diameter cut from a bigger piece were weighed $\left(W_{d_{o}}\right)$ and put into the 24-well plate. Next, each sample was poured with 1 mL of phosphate-buffered saline (PBS, pH 7.4). All of the materials were incubated for 24 h at 37 °C under constant shaking (100 rpm). Afterward, the buffer was withdrawn, the samples were gently blotted with a piece of paper and weighed again (*W*_s_). The swelling degree (SD) was calculated based on eq 1:

1 $$SD\;\left(\%\right)=\frac{W_{s}-W_{d_{o}}}{W_{d_{o}}}\cdot 100\%$$

##### 2.3.1.3 Degradation Study

Materials (D2, D3, D4, E2, E3, E4, F2, F3, F4, I2, J2, K2, and L2) cut into disks (1 cm in diameter) were placed into the wells of a 24-well plate, and 1 mL of DI was added to each sample. The systems were incubated for 2 h at 37 °C while being shaken (100 rpm). After that time, the water was removed, the specimens were gently pressed to the paper to withdraw the excess liquid, and weighed, giving the *W*_0_. Then, 1 mL of PBS was introduced to each well with the material, and the systems continued to be incubated under the previously applied conditions (37 °C, shaking at 100 rpm). At specific time points of 24 h, 48 h, 72 h, and 144 h, the materials were reweighted (*W*_t_), and the buffer was exchanged. Changes in mass occurring over time were established using the following eq 2:

2 $$\mathrm{Mass change}\;\left(\%\right)=\frac{W_{t}}{W_{o}}\cdot 100\%$$

##### 2.3.1.4 Porosity Measurements

A helium pycnometer (Micromeritics, AccuPyc II 1340) was used to ascertain the density of the selected materials. Further, the mercury porosimetry technique (PoreMaster 33, Quantachrome Inc.) was used to determine the porosity in the range from 7 nm to approximately 80 μm and parameters such as total pore volume, median, and mode of the selected flake-like materials (D3, D4, E4, F4, K2, L2). Three neurosurgical hemostatic devices, TachoSil, Clinisponge, and Reoxcel, were also subjected to exact measurements.

#### 2.3.2 Chit-VANC Particles Verification

The generated objects were imaged using scanning electron microscopy (SEM, HITACHI S-4700) after being cross-linked into a model hydrogel system composed of collagen, chitosan, and lysine-modified hyaluronic acid.

#### 2.3.3 Inclusive Complexes TMZ-βCD Analysis

The product of TMZ with βCD complexation was characterized based on spectra obtained by UV–vis (Spectrophotometer Hitachi U-2900), nuclear magnetic resonance (NMR, Bruker Avance III 600 spectrometer), and Fourier transform infrared (FTIR, Thermo Fisher Scientific Nicolet IR200) spectroscopies, as well as SEM microphotographs.

#### 2.3.4 Multifunctional Flake-Like Composites Examination

##### 2.3.4.1 Vancomycin Encapsulation Efficiency (EE) Evaluation

The encapsulation efficiency of vancomycin in selected materials (D3-VTC, D4-VTC, E4-VTC, F4-VTC, K2-VTC, and L2-VTC) was established by quantifying the antibiotic content not incorporated within the material. The measurements were performed using a spectrofluorimetric method described in the literature,^33^ with the procedure modified and optimized to suit the requirements of this study. After each flake was rinsed, the DI was collected, mixed with methanol in a 1:9 ratio, and subsequently analyzed by using a spectrofluorometer with a fluorescence signal at 335 nm following excitation at 280 nm. The unencapsulated VANC amounts were determined from the calibration curve and recorded for samples with known vancomycin concentrations treated similarly.

##### 2.3.4.2 Preliminary Assessment of Ca^2+^ Ions

The preliminary assessment of Ca^2+^ content in the tested materials was conducted spectrofluorimetrically using a determination with calcein.^34^ Discs of various materials (D3-VTC, D4-VTC, E4-VTC, F4-VTC, K2-VTC, and L2-VTC) were weighed and placed into a 24-well plate. To each replicate, 2 mL of DI was added, and the materials were incubated for 30 min at 37 °C while being shaken (100 rpm). Following incubation, 1 mL of the withdrawn liquid was mixed with an equal volume of calcein solution (0.5 mg/mL) in 2 M KOH. Fluorescence spectra, with a maximum emission at 540 nm, were recorded, and the quantitative analysis of Ca^2+^ was performed using a calcein standard curve.

##### 2.3.4.3 EE of TMZ-βCD Examination

Encapsulation efficiency was calculated by referring to the content of TMZ-βCD complexes that were not included in the hydrogel-based flakes of different types (D3-VTC, D4-VTC, E4-VTC, F4-VTC, K2-VTC, and L2-VTC). It was evaluated by measuring the absorption spectra of the solution in which the samples were incubated for 24 h during the loading stage, and the DI solutions were collected after material rinsing. The amount of the drug was assessed based on the calibration curve for TMZ and the absorption maximum at 330 nm.

##### 2.3.4.4 In Vitro Release Study of TMZ

Materials containing TMZ-βCD complexes (D3-VTC, D4-VTC, E4-VTC, F4-VTC, K2-VTC, and L2-VTC) were placed into glass vials, immersed in 4 mL of PBS (pH 7.4), and incubated at 37 °C with shaking at 100 rpm. To evaluate the amount of released drug at specific time points (0.5, 1, 2, 4, 8, 24, 48, 72, and 144 h), 2 mL of PBS was withdrawn from each sample and replaced with an equal volume of fresh buffer. The collected samples were then returned to the incubator under the same conditions (37 °C, shaking) to allow for drug hydrolysis. Once no absorption signal at 330 nm was detected, the UV–vis spectra were measured, and the peak at 267 nm was used to quantify the released drug, referencing the calibration curve for TMZ after hydrolytic degradation (the 5-aminoimidazole-4-carboxamide (AIC) form). The molar extinction coefficient (ε) of AIC was determined in PBS 7.4, and was found to be 11,051 ± 77 L · mol^–1^ · cm^–1^ at 267 nm.

To evaluate the kinetics of the release profiles, three mathematical models were fitted to the experimental data as follows (eqs 3–5):

First order^35^

3 $$\frac{M_{t}}{M_{\infty }}=F_{\max}\cdot \left(1-e^{-kt}\right)$$

where *M_t_* —the amount of the drug released after *t* time, *M*_∞_—total drug content, k—release constant, *t*—time, *F*_max_—maximal substance fraction that was released.

Weibull 3 ^35^

4 $$\frac{M_{t}}{M_{\infty }}=F_{\max}\cdot \left(1-e^{-t^{b}/a}\right)$$

*a*—scale parameter, *b*—shape parameter.

Double-exponential^36^

5 $$\frac{M_{t}}{M_{\infty }}=A_{1}\cdot \left(1-e^{-k_{1}t}\right)+A_{2}\cdot \left(1-e^{-k_{2}t}\right)$$

*A*_1_, *A*_2_—drug fractions released during the rapid release phase (*A*_1_) and sustained release (*A*_2_); k_1_, k_2_—rate constants of those phases.

### 2.4 Antibacterial Evaluation In Vitro

A series of hydrogel materials was subjected to antibacterial activity against *Staphylococcus aureus* (ATCC 25923) and *Escherichia coli* (ATCC 25922). The developed multifunctional composites (D3-VTC, D4-VTC, E4-VTC, F4-VTC, K2-VTC, and L2-VTC) were prepared in a 24-well plate and sterilized with UV radiation for 20 min. For the tested commercial materials (TachoSil, Clinisponge, and Reoxcel), discs with a diameter similar to that of the hydrogel samples were cut. Both bacterial strains were grown in Mueller-Hinton II (MHII) Broth until the mid-logarithmic phase of growth was reached. After that, the bacteria were spread on the surface of the MHII agar medium in Petri dishes. Hydrogel-based discs were placed on the surface of such prepared dishes, and 100 μL of PBS buffer was gently placed on them (in the case of Reoxcel, the volume of PBS was 20 μL). The plates were left at room temperature for 30 min and placed in an incubator at 37 °C overnight. We have observed the hampering of bacterial zones only in the case of culturing of *S. aureus*.

### 2.5 Biological Study

#### 2.5.1 Cell Lines and Human Blood Cells Used in Experiments

U-251 MG human glioblastoma cells (ECACC 09063001) were purchased from the European Collection of Authenticated Cell Cultures. Human umbilical vein endothelial cells (HUVECs; used in experiments to the fourth-fifth passage) were obtained from PromoCell, Germany. HepG2 human hepatocellular carcinoma cells (ATCC HB-8065) were purchased from the American Type Culture Collection.

Citrated blood from healthy volunteers was purchased from the Regional Center for Blood Donation and Treatment in Kraków. Whole blood was used for the hemolysis assay. Human peripheral blood mononuclear cells (PBMCs) were isolated using a Ficoll-Paque Plus density gradient (GE Healthcare, Chicago, IL, USA), and they were used after thawing. In compliance with confidentiality requirements for human subjects, the Regional Center for Blood Donation and Treatment deidentified all blood materials.

#### 2.5.2 Hydrogel Flake Preparation for Direct Interaction with Cancer and Endothelial Cells

Before the experiment, hydrogel flakes of various sizes were sterilized under UV light for 15 min, washed twice with PBS, and incubated for 30 min in the appropriate cell culture medium: (1) Dulbecco’s modified Eagle medium GlutaMAX (DMEM, 4.5 g/L glucose; Thermo Fisher Scientific) for U-251 MG cells or (2) ready-to-use endothelial cell growth medium (ECGM; PromoCell) for HUVEC cells. After incubation, the medium was removed, and the materials were transferred onto the cell cultures.

#### 2.5.3 Cell Viability

U-251 MG cells were seeded at a density of 1 × 10^4^ cells per well in a 48-well plate in 0.4 mL of DMEM (4.5 g/L glucose), 10% FBS (fetal bovine serum, Thermo Fisher Scientific), 10,000 U/mL penicillin, and 10,000 μg/mL streptomycin (PEST, Thermo Fisher Scientific)—Day 0. After overnight attachment of the cells to the plate surface, the 5 mm-diameter flake-like materials were transferred onto the cells—Day 1. The cells were incubated with the materials for either 3 or 7 days. Subsequently, cell viability was assessed using the AlamarBlue assay (Thermo Fisher Scientific), which was performed according to the manufacturer’s instructions. Fluorescence intensity was measured using a Synergy H1 Hybrid microplate reader with Gen5 software, version 2.00.18 (BioTek Instruments). Cell viability was expressed as a percentage relative to the control (cells cultured without the material).

#### 2.5.4 Cell Cycle Evaluation

U-251 MG cells were seeded in a 6-well plate at a density of 3 × 10^5^ cells in 3 mL of DMEM containing 10% FBS with PEST. The next day, the chosen materials made from 1260 μL of a specific sol were transferred above the cells. After 74 h of incubation, cells were trypsinized (Trypsin, BioWest) and fixed with 70% ethanol (EtOH)—1 mL of cell suspension in PBS (Thermo Fisher Scientific) was transferred to 5 mL of cold 70% EtOH. Fixed cells were stored at 4 °C for at least one night, then washed with PBS and stained with FxCycle PI/RNase Staining Solution (Thermo Fisher Scientific). The cell cycle was analyzed by flow cytometry (BD FACS Calibur with BD CellQuest Pro Software) in the FL2-H channel after aggregate removal in an FSC vs FL2-A dot plot (gating strategy presented in Figure S1A).

#### 2.5.5 Hydrogel Proinflammatory Properties Evaluation Based on VCAM-1 Measurement

HUVEC cells were seeded at a density of 1.5 × 10^5^ cells in 3 mL on a 6-well plate in ready-to-use ECGM with PEST. After overnight cultivation, the materials were transferred above the cells for 24 h. Subsequently, the cells were detached with Accutase (Thermo Fisher Scientific) and stained with CD106 (VCAM-1) Monoclonal Antibody conjugated with Alexa Fluor 488 eBioscience InvivoGen) to visualize VCAM-1. Mouse IgG1 kappa Isotype Control (P3.6.2.8.1), Alexa Fluor 488, and eBioscience (InvivoGen) were used as the isotype control. TNF at a concentration of 10 ng/mL was used as the positive control for VCAM-1 expression on the cell surface. Mean fluorescence intensity was measured as VCAM-1 content in the FL1-H channel after the exclusion of dead cells based on PI staining (this dye internalizes only into dead cells)—the gating strategy is presented in Figure S1B.

#### 2.5.6 Hemostatic Characterization of Materials

Citrated whole blood from a healthy donor was obtained from the Regional Center for Blood Donation and Treatment in Kraków. Before the experiment, the blood and the test materials (prepared in specified dimensions) were equilibrated to 37 °C. To neutralize the anticoagulant effect of sodium citrate, 25 mM CaCl_2_ was added to the blood. Immediately afterward, 0.5 mL of the recalcified blood was transferred to a 6-well plate into an empty well or directly onto the materials already placed in the wells. The blood was then incubated with the materials for 15 min at 37 °C. Following the incubation, the materials were gently rinsed with 3 mL of distilled water dispensed along the side wall of the well to minimize disruption of surface-adhered cells. The rinse solution containing detached erythrocytes was collected into a fresh Falcon tube and diluted with 3 mL of distilled water. The amount of hemoglobin released from lysed erythrocytes was quantified spectrophotometrically at 540 nm. Further samples were rinsed once with 1 mL of PBS and then fixed with 2.5% glutaraldehyde for 75 min. After fixation, the samples were dehydrated using a graded ethanol series (60%, 70%, 80%, and 90% for 5 min each, followed by 100% ethanol for 15 min) and were allowed to air-dry overnight. The samples were next stuck on carbon tape, sputtered with gold, and imaged using scanning electron microscopy (SEM, HITACHI S-4700).

#### 2.5.7 Extract Collection and Their Incubation with Cells

Hydrogel flakes in 12-well plates were washed twice with PBS and incubated with DMEM 4.5 g/L glucose and PEST for 30 min. Subsequently, 1 mL of fresh DMEM 4.5 g/L glucose and PEST was added to each well, incubated for 4 h, and collected as the extract obtained after 4 h of material incubation with DMEM. This procedure was repeated on days 1, 2, 3, or 6, replacing the medium with a fresh one each time.

#### 2.5.8 Hemolysis Assay

Hemolysis was performed according to a standard protocol. Briefly, the experimental mixture was prepared by adding (1) 800 μL of PBS, (2) 100 μL of hydrogel flake extract, and (3) 100 μL of 10-times diluted whole blood. All samples were incubated at 37 °C with agitation every 15 min for 3 h. DMEM 4.5 g/L glucose with PEST served as the negative control (representing hemolysis in intact red blood cells, RBCs), and 0.1% Triton X-100 as the positive control (representing total RBC hemolysis). After incubation, RBCs were centrifuged at 100 × *g* for 5 min. Then, 100 μL of the supernatant was collected, and the Drabkin reaction with free hemoglobin was performed in a 96-well plate. Absorbance was measured at 540 nm, and its intensity was directly proportional to the degree of hemolysis.

#### 2.5.9 PBMC and HepG2 Cell Viability

PBMC, after thawing, were seeded at a density of 1 × 10^5^ cells in 100 μL of RPMI-1640 medium supplemented with 20% FBS and PEST. Immediately after seeding, 10 μL of hydrogel extract was added directly to the culture medium. After 24 h of incubation, PBMC viability was measured using the ATPlite firstep Luminescence Assay System (Revvity), following the manufacturer’s protocol. Luminescence intensity was measured by using a microplate reader.

HepG2 cells (at a density of 5 × 10^3^ cells) were seeded in 100 μL of DMEM (1 g/L glucose) supplemented with 10% FBS and PEST. On the second day, 10 μL of hydrogel extract was added directly to the culture medium, and HepG2 viability was measured 48 h later using the MTT assay according to the standard protocol.

### 2.6 Statistics

The gathered results are presented as mean values ± standard deviation based on a minimum of three replicates. Student’s *t*-test was applied to assess significance levels defined as follows: *p* ≤ 0.05 (*), *p* ≤ 0.01 (**), and *p* ≤ 0.001 (***). For biological studies, multiple *t*-tests were performed using GraphPad Prism 10.4. Statistical significance was confirmed when *p* ≤ 0.01 (*).

## 3 Results and Discussion

### 3.1 Biopolymeric Matrix Design

The proper design of the material intended to be implemented in the tumor bed after resection is crucial. This system must exhibit beneficial therapeutic action and, most importantly, ensure patients’ safety. The comprehensive material optimization may lead us to a product that meets the necessary criteria for successfully performing its function.

#### 3.1.1 Determination of −NH_2_ Groups in Biopolymeric Sols of Different Composition

The initial step in producing materials obtained via genipin-driven chemical cross-linking—an agent that utilizes amino groups to form a three-dimensional biopolymeric network—involved the quantification of available −NH_2_ residues. The corresponding data for 12 biopolymeric sols (A–L), each characterized by different gelatin:chitosan:hyaluronic acid modified with lysine (Gel:Chit:HA_mod_) weight ratios, are presented in Figure 2A. The −NH_2_ content ranged from approximately 47 to 60 μmol per milliliter of sol. The highest concentrations were observed in sols I, J, K, and L, which contained 50% chitosan—a polycationic biopolymer—and in sols D and E, with a 40% chitosan content. Notably, the experimentally determined −NH_2_ concentrations correspond well with theoretical predictions (Figure 2B), which were assessed based on the chemical structures of chitosan (with a 70% degree of deacetylation), hyaluronic acid, and gelatin.^37,38^

**Figure 2:**
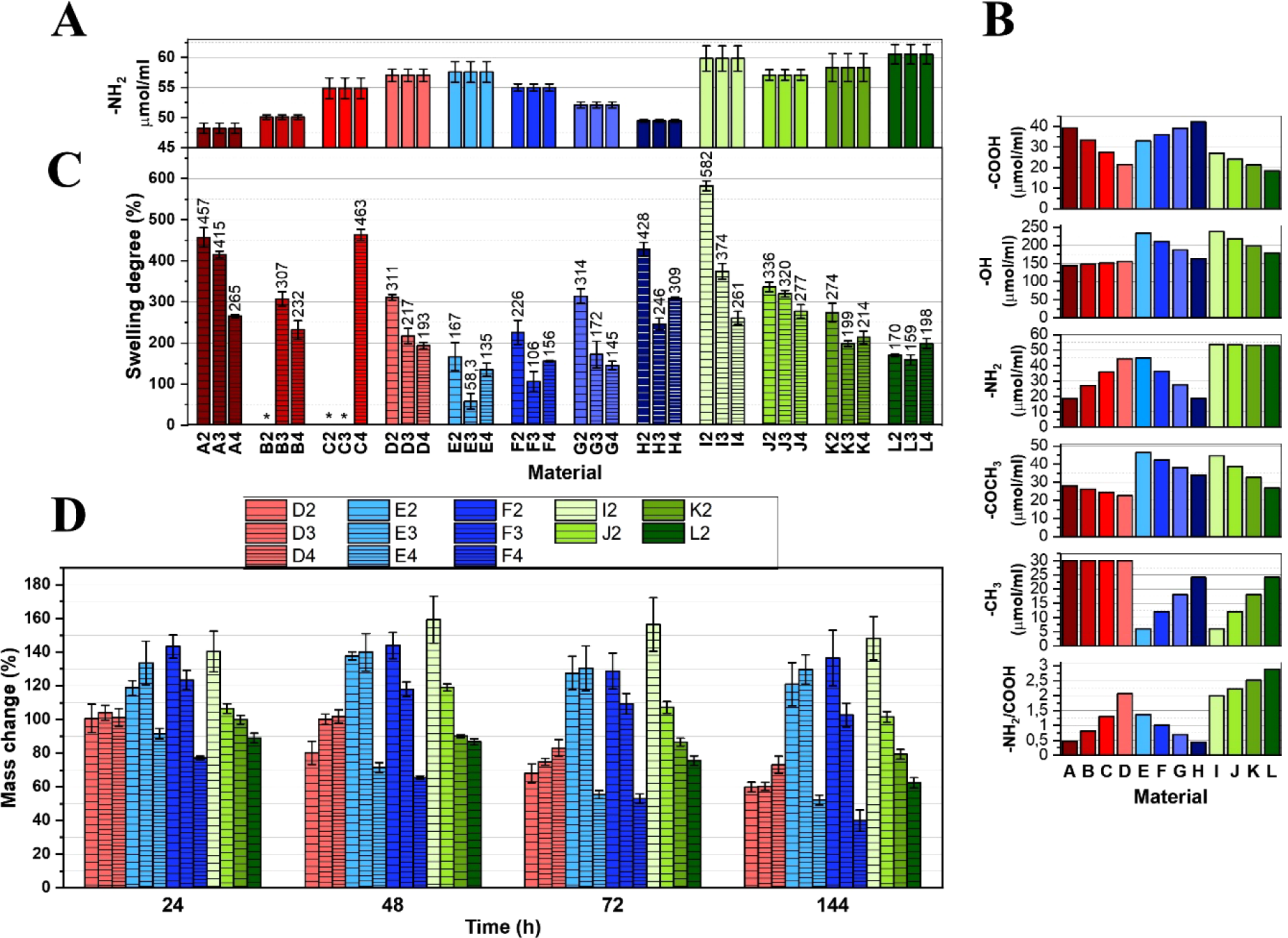
Results of: (A) determination of the content of amine groups in sols composed of biopolymers with different Gel:Chit:HA_mod_ mass ratios; (B) calculations of the theoretical share of specific functional groups in these systems; (C) the degree of swelling of systems obtained using the mentioned sols and three different concentrations of genipin; (D) changes in the mass of selected hydrogel-based materials observed during the degradation experiment.

#### 3.1.2 Swelling Evaluation

In principle, systems with a higher content of primary amino groups (−NH_2_) are expected to undergo more effective cross-linking, resulting in matrices with improved structural integrity and a further lower swelling degree (SD) than systems with fewer reactive sites. However, cross-linking efficiency is not solely determined by the −NH_2_ level. The complexity of the proposed compositions and the interactions among the biopolymeric sol components significantly affect the final network structure. Experimental SD data are presented in Figure 2C. A general trend can be observed: in systems containing 50% of a single polymer (except for C4), a higher −NH_2_/–COOH ratio corresponds to a lower SD. Nevertheless, this trend is not universal, as specific interactions between side groups may hinder proper network formation, leading to partial or complete material disintegration (e.g., B2, C2, and C3), even in systems with theoretically sufficient amine content (Figure 2A).

As can be seen, when the only variable is the genipin (GEN) amount, the highest SD values are noted for systems with the lowest cross-linking agent levels (A2, D2, E2, F2, G2, H2, I2, K2). Within individual series (e.g., A2–A4), increasing GEN concentration (from 2.65 to 6.63 mM) generally reduces the SD, although this trend is not consistent across all compositions (especially when comparing the same sols but treated with 4.64- and 6.63-mM GEN concentrations). The reason for such a response of the material is that at higher concentrations of GEN, its polymerization induced by free oxygen radicals is possible.^39^ Polymerized genipin units can act as spacers between biopolymer chains, forming looser internal structures with an increased porosity. In such a situation, genipin may act as a plasticizer,^40^ enhancing the hydrogel’s ability to absorb water and increasing the materials’ SD.

For degradation studies, representative formulations comprising 50% gelatin (D2–D4), modified hyaluronic acid (E2–E4, F2–F4), or chitosan (I2, J2, K2, and L2) were selected. While materials rich in gelatin and HA_mod_ exhibited a consistently reduced swelling capacity, those with high chitosan content showed a clear composition-dependent response in SD values.

Overall, materials with tunable physicochemical properties can be developed by playing with cross-linking agent concentration and biopolymer ratios. The experimental monitoring of swelling degree provides valuable insight into the structural and functional materials’ potential for implantation into brain applications.

#### 3.1.3 Degradation Study

To predict the behavior of the materials under simulated physiological conditions (37 °C, pH 7.4), selected systems were subjected to a degradation study; the corresponding results are illustrated in Figure 2D.

After 24 h of the experiment, an increase in the mass of most of the analyzed materials was observed toward their initial value (m_0_). The most significant increases were noted for the E2 (118%), E3 (133%), F2 (143%), F3 (123%), and I2 (140%) systems (for statistical analysis, see Figure S2). This phenomenon results from the high share of hydrophilic groups and low concentrations of the cross-linking agent (2.65 mM for E2, F2, and I2; 4.64 mM for E3 and F3). Consequently, −NH_2_ residues not used in the cross-linking process remained available for interacting with water molecules. The E2, E3, F2, I2, and J2 systems demonstrated the greatest stability under experimental conditions, characterized by the slightest mass fluctuations. After the initial swelling, the mass change in these materials did not exceed 5%. Approximately 60% of the initial mass remained in the case of materials cross-linked with the two lower concentrations of genipin (2.65 or 4.64 mM), where 50% of the matrix consisted of gelatin (systems D2 and D3, 60%) or chitosan (system L2, 62%). The degradation of D2 and D3 proceeded slightly faster—the mass loss reached up to 25% at subsequent time points—compared to L2, where changes ranged from a few to 13%. This outcome is probably related to the faster degradation of gelatin under the experimental conditions (37 °C, pH = 7.4, shaking). Increasing the genipin concentration stabilized the structure^41^ and slowed down the degradation process in the D4 material compared to D2 and D3, resulting in a 10% growth in the remaining material mass after 144 h. The lowest final mass values (after 144 h) were observed for the materials that were probably the most highly cross-linked, containing 50% HA_mod_–F4 (40%) and E4 (52%). The highest concentration of genipin (6.63 mM) was used in these systems. We suspect that combining many hydrophilic groups with intensive cross-linking led to the formation of internal stresses and microcracks, which could have contributed to accelerated degradation compared to the other systems. Additionally, a slightly faster mass loss was perceived in the F3 and F4 systems compared to their counterparts, which contained slightly less gelatin—E3 and E4. In the case of materials containing 50% chitosan, the increase in the share of HA_mod_ (for I2 and J2 systems) promoted an increase in their mass due to the retention of solvent molecules. In turn, a higher content of gelatin (systems K2 and L2) contributed to the acceleration of degradation.

All tested materials showed high stability, indicating their favorable potential for gradual degradation, facilitated absorption, and excretion of degradation products by the body.^42^ Based on the results, the D3, D4, E4, F4, K2, and L2 systems were selected for further characterization due to their most favorable swelling properties and degradation profiles corresponding to the intended application.^14^

#### 3.1.4 Porosity Measurements

The porous structure of a multifunctional implantable into the brain material is crucial, since it plays a key role during the diffusion of gases and nutrients and exudate absorption. Furthermore, open pores facilitate the migration and aggregation of blood morphological elements responsible for the initiation of the clotting process. At the same time, the system must provide mechanical stability, ensuring material integrity at the implementation site under physiological conditions.^43^ The literature describes materials with high porosity and pore sizes of approximately 500 μm,^44^ allowing for neuron infiltration and further neuronal tissue growth. On the other hand, membranes with pores of 0.5 μm are used as a BBB model.^45^

To determine the functional properties of our systems, we assessed their application potential as a result of a comparative analysis with commercially available hemostatic agents commonly used during neurosurgical procedures. The porous structure was evaluated using mercury porosimetry, which determined parameters such as total porosity, open pore volume, and pore diameter distribution (Table 2). The porosity of all tested biopolymeric matrices falls within or near the range observed for commercial hemostatic materials (Reoxcel, TachoSil, Clinisponge), i.e., 50–88%. The highest values (∼88%) were recorded for K2, D3, and D4, while the lowest values were observed in systems containing 50% HA_mod_ (E4:59%, F4:46%). The dominant pore size range of 5–80 μm confirmed the macroporous character of all synthesized materials. Furthermore, the determined pore mode has proven comparable across fabricated systems (∼52–59 μm), aligning with Clinisponge. The median pore size differed more significantly between samples: D3, D4, E4, and K2 exhibited dominant pores with diameters of 42.5–45.9 μm, whereas lower values were noted for L2 (36.6 μm) and F4 (23.1 μm). Total pore volume ranged from 0.443 to 0.757 cm^3^/g for F4, E4, and L2; was several times higher for D3 and D4 (2.389 and 2.866 cm^3^/g, respectively); and peaked for K2 (3.1 cm^3^/g). Notably, all values remained similar to the commercial hemostatic systems (0.53–4.09 cm^3^/g).

**Table 2:** Data Regarding Porosity, Total Pore Volume, as Well as Mode and Median Pore Diameters in the Range of 5–100 μm for Materials with Different Biopolymeric Compositions and Genipin Crosslinking Agent Concentration

		Pores diameter [μm]		
Type of material	Porosity [%]	Mode	Median	Total pores volume (open pores) [cm^3^/g]
D3	87.08	58.85	45.94	2.389
D4	88.38	55.43	43.88	2.866
E4	59.04	59.25	44.17	0.519
F4	46.33	56.30	23.11	0.443
K2	88.59	52.39	42.50	3.100
L2	68.53	53.30	36.60	0.757
Reoxcel	49.90	28.30	28.70	0.530
Tachosil	88.18	27.10	21.90	4.090
Clinisponge	72.15	52.65	48.06	1.904

Differences in porosity can arise from variations in matrix composition, cross-linking agent concentration, and the distribution of functional groups. These factors affected the cross-linking process and the spatial organization of polymer chains, ultimately shaping the internal architecture of the materials. It seems that the noted similarity of the properties of all the obtained materials to commercial hemostatic systems and the substantial share of pores in the 5–80 μm range would ensure the flow of platelets with sizes of 1–2 μm as well as erythrocytes, which are 6–8 times larger and necessary for the clotting process.^46^

### 3.2 Chit-VANC Particles Verification

To minimize the incidence of infections after surgical procedures and enhance the therapeutic efficacy of our multifunctional flakes, vancomycin (VANC), an antibiotic effective against Gram-positive bacteria, was incorporated as one of the functional elements.^24^ VANC was introduced into the system as an ionically cross-linked chitosan-TPP particle (chit-VANC) formulation, which acts as an antibacterial support at the implantation site. Encapsulating VANC molecules into chitosan-based carriers was designed to ensure uniform particles’ embedding within the biopolymeric matrix and their stable integration with other material components.

The obtained chit-VANC particles were imaged upon incorporation into a model system composed of collagen, chitosan, and HA_mod_, which were selected to improve the carriers’ visibility under a scanning electron microscope. The microphotographs (Figure 3) revealed two particle size populations—smaller ones with a diameter of ∼0.5 μm and twice-as-big carriers (∼1 μm). The formed structures were clearly observable when attached to collagen fibrils due to successful cross-linking with genipin. The obtained microphotographs confirmed that the chit-VANC particles can be implemented into the hydrogel matrix via chemical cross-linking without undergoing fragmentation.

**Figure 3:**
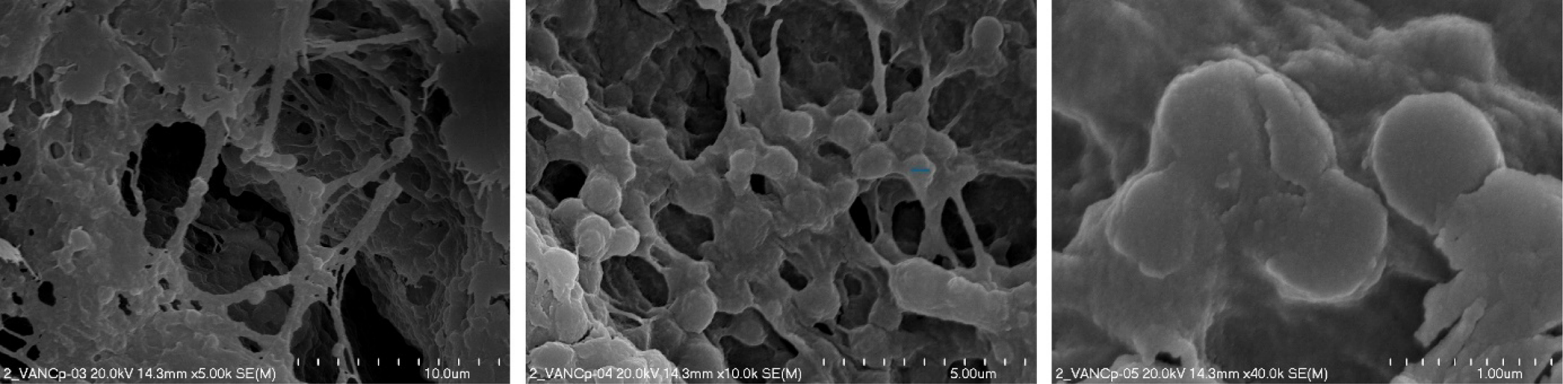
SEM microphotographs of Chit-VANC particles in a biopolymeric model system.

### 3.3 Inclusive Complexes, TMZ-βCD Analysis

To impart the systems with cytotoxic properties against residual glioma cells remaining in the postoperative tumor bed and thus minimize the risk of tumor recurrence, it was decided to use temozolomide (TMZ) as an active ingredient in the obtained biopolymer flakes. Due to the limited solubility of TMZ and its low hydrolytic stability in the conditions of forming hydrogel matrices, the approach was chosen to introduce the drug in the form of a guest–host inclusion complex with β-cyclodextrin (TMZ-βCD, Figure 4A). Boroushaki et al.^47^ demonstrated that the formation of TMZ-βCD inclusion complexes is thermodynamically favorable, with van der Waals interactions being the dominant driving force behind their assembly. Furthermore, they showed that TMZ displaces water molecules from the β-cyclodextrin cavity and that its structure fits well into the hydrophobic interior of the host molecule. These findings are consistent with experimental data reported in the literature.^31,48^ Moreover, it has been demonstrated that the formation of the inclusion complex extends the half-life of TMZ from 1.2 h (free TMZ) to 5.5 h (TMZ-βCD) at pH 7.4. This represents a significant improvement, as one of the major challenges in both research and clinical use of TMZ is its low hydrolytic stability.^48^

**Figure 4:**
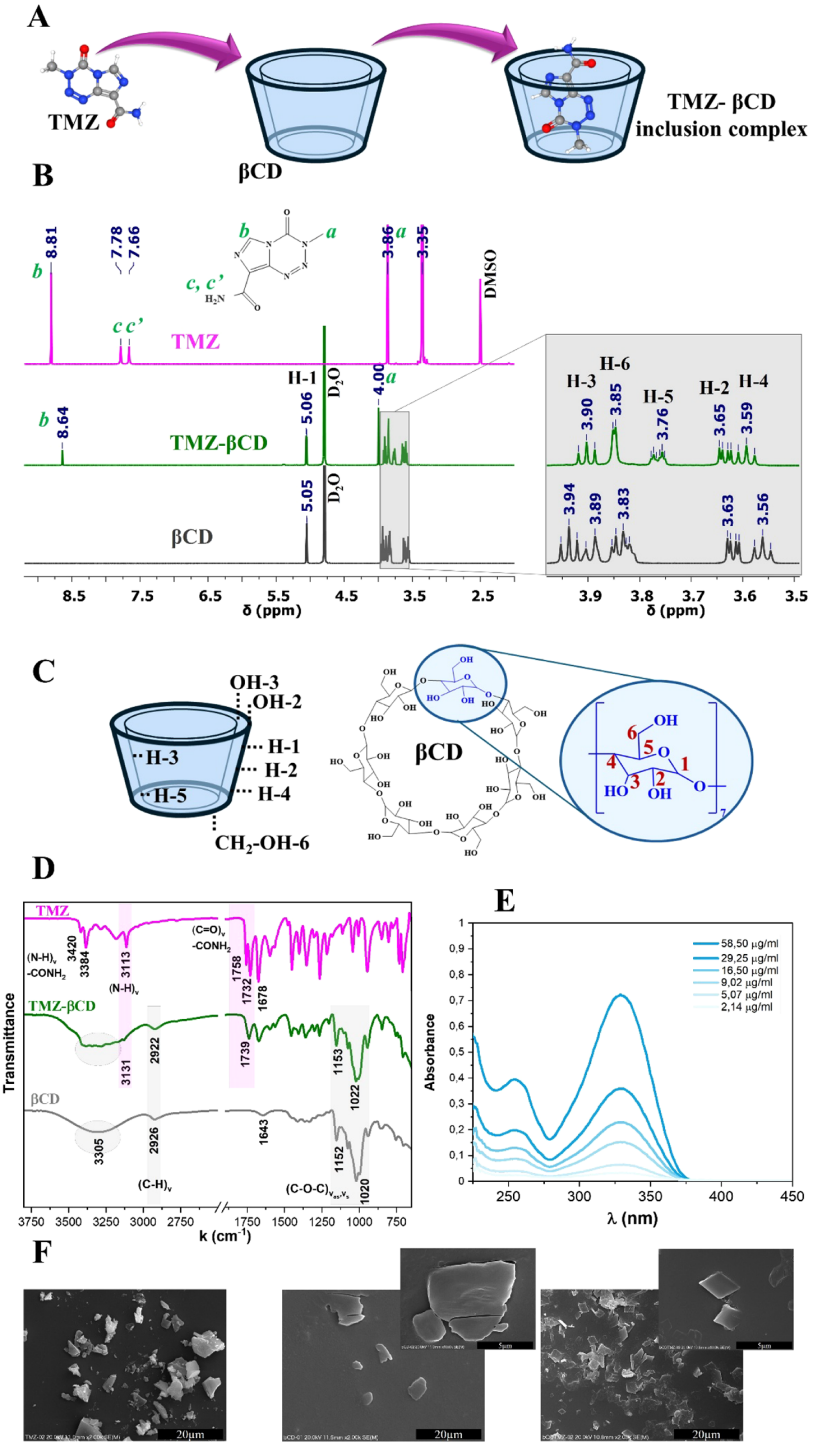
Schematic representation of the host–guest inclusion complex between temozolomide (TMZ) and β-cyclodextrin (β-CD) (A); ^1^H NMR spectra of TMZ, native βCD, and the formed TMZ-βCD complex (B), along with chemical structure and proton assignments (C); FTIR spectra comparing the complex and its components (D); UV–Vis analysis for TMZ-βCD (E); SEM micrographs showing morphological changes upon complexation (F).

The product of loading βCD with TMZ was characterized by using ^1^H NMR spectroscopy (Figure 4B). The presented data for βCD and TMZ-βCD were registered in D_2_O to maintain complex stability, since using organic solvents such as DMSO-d6 (which was used for the TMZ spectrum) could have resulted in a complex breakup.

^1^H NMR spectra (Figure 4B) proved the presence of both components, forming the inclusion complex. Compared with the data gathered for free βCD, the characteristic proton signals in the TMZ-βCD complex (Figure 4C) exhibited noticeable chemical shift changes (Table 3). Differences in chemical resonance location were observed for all proton signals. Still, the most significant upfield displacements (toward lower δ values) were noted for H3 (from 3.94 ppm for free β-CD to 3.90 ppm for the complex), located inside the cavity near the wider rim; H5 (3.83 ppm for β-CD, 3.76 ppm for TMZ-β-CD), positioned deep inside the hydrophobic cavity, close to the narrower rim; and H6 (3.89 ppm for β-CD, 3.85 ppm for TMZ-β-CD), situated on the outer surface near the OH-6 group. These three shifts supported the previously postulated^47^ entry of the TMZ molecule from the wider rim, deep inclusion into the β-CD cavity, and reaching the cyclodextrin’s lower (narrower rim) part. Additionally, characteristic resonances marked as a and b in the complex spectrum at δ = 4.00 ppm (3H, CH_3_) and δ = 8.64 ppm (1H, CH) affirmed the presence of TMZ in the system.^49^

**Table 3:** Chemical Shifts’ Changes Observed in the ^1^H NMR Spectra of β-Cyclodextrin and the TMZ-βCD Complex

βCD protons	δ in free βCD (ppm)	δ in TMZ-βCD complex (ppm)	Δδ (ppm)
H1	5.05	5.06	–0.01
H2	3.63	3.65	–0.02
H3	3.94	3.90	+0.04
H4	3.56	3.59	–0.03
H5	3.83	3.76	+0.07
H6	3.89	3.85	+0.04

Comparative analysis of the FTIR spectra obtained for the TMZ-βCD inclusion complex and its individual components (Figure 4D) may offer valuable insights into the intramolecular interactions and provide evidence for complex formation. In the spectrum of the complex, characteristic signals originating from the β-cyclodextrin framework and functional groups of the TMZ molecule were observed. Notably, several of these bands exhibit differences in shape, intensity, or position when compared to the spectra of the native components. This suggests alterations in the molecular environment due to the inclusion of TMZ within the hydrophobic cavity of β-cyclodextrin. Among the signals attributed to β-cyclodextrin, the broad O–H stretching vibration was detected around 3305 cm^–1^, while the C–H stretching band shifted slightly from 2926 cm^–1^ to 2922 cm^–1^.^50^ Minor changes were also observed in the vibrations corresponding to the C–O–C framework (1153 and 1022 cm^–1^). In the same spectrum, the −CONH_2_ group from TMZ was confirmed by a characteristic band (C=O) at 1739 cm^–1^ and a significantly diminished N–H stretching vibration at 3131 cm^–1^.^51^

In the collected UV–vis spectrum for TMZ-βCD (Figure 4E), a characteristic maximum at 330 nm corresponding to the imidazotetrazine ring was visible, which indicated the preservation of TMZ structural integrity and confirmed the lack of hydrolytic degradation under the conditions used during complex formation.

For qualitative analysis and additional verification of the complex formation, scanning electron microscopy (SEM) imaging was employed. The SEM micrographs of the substrates—both temozolomide (Figure 4F on the left) and β-cyclodextrin (Figure 4F in the middle)—revealed irregular structures with rough surfaces, organized in heterogeneous aggregates and irregular geometric, smooth, round-edged objects, respectively. In contrast, the SEM image of the complexation product (Figure 4F on the right) displayed pronounced morphological transformation, resulting in the emergence of more uniform, square- or rhomboid-shaped crystals with smoother surfaces. The morphological differences observed between the micrographs of the substrates and the product may result from guest–host interactions, indicating the formation of an inclusion complex. Such changes in micromorphology are consistent with the formation of inclusion complexes and have been reported in the literature for various β-cyclodextrin-based systems.^31,52^

### 3.4 Multifunctional Flake-Like Composites Examination

#### 3.4.1 Vancomycin Encapsulation Efficiency (EE) Evaluation

The encapsulation efficiency of vancomycin (VANC EE) in the resulting biopolymer-based flakes ranged from 66.3% to 84.4% (Table 4). The highest VANC EE values were observed in systems synthesized with the highest genipin (GEN) concentrations (D4–80.7%, E4–84.4%, F4–82.1%) as well as for materials with a 50% share of chitosan and the lowest cross-linking agent content (K2–76.4%, L2–79.3%). The observations imply that interactions occurring between components of the biopolymeric sol significantly influence the materials’ internal architecture and further affect the encapsulation efficiency of compounds incorporated as a chitosan particle formulation. Similarly, the swelling behavior across materials reflected the interplay among biopolymeric chains. Notably, although adding VANC encapsulated in chit-VANC particles could have disrupted the main biopolymeric network, the tested systems exhibited enough flexibility to integrate the particles within the matrix. A lack of observed significant impact on particle embedment (as evidenced by comparable results for E4 vs F4 and K2 vs L2) pointed out that the primary encapsulation mechanism is associated with cross-linking with genipin and is not dependent on matrix composition in the tested range. Altogether, the results indicate that the method of antibiotic incorporation into the system is effective and does not impair materials’ formation or integration of the particles.

**Table 4:** Vancomycin Encapsulation Efficiency and Quantification of Ca^2+^ Ions Released from the Tested Systems after 30 min, Expressed Per Milligram of Material

Type of material	Vancomycin encapsulation efficiency (%)	Mass of calcium per 1 mg of the system (μg)
D3-VTC	66.3 ± 3.2	67 ± 2
D4-VTC	80.7 ± 1.4	61 ± 2
E4-VTC	84.4 ± 2.0	36 ± 1
F4-VTC	82.2 ± 2.8	36 ± 1
K2-VTC	76.4 ± 1.8	60 ± 6
L2-VTC	79.3 ± 1.1	45 ± 1

#### 3.4.2 Preliminary Assessment of Ca^2+^ Ions Presence

One of the desired functionalities in the designed materials is their hemostatic potential. Since the role of calcium ions (Ca^2+^) is essential and well-established during initiation, as well as regulating the blood coagulation cascade, their incorporation into the systems was pursued.^53^ Due to the rapid activation of the coagulation process, the availability of Ca^2+^ ions at its early stages is considered particularly important to influence the efficiency of bleeding control.

The determined amounts of Ca^2+^ ions released within 30 min per 1 mg of the synthesized materials are gathered in Table 4. The quantification was performed using complexation with calcein since Ca-calcein complexes exhibit higher fluorescence in an alkaline environment than the fluorophore alone.^54^

A clear relationship can be seen between the content of Ca^2+^ ions and the material’s porosity. The largest amounts of released Ca^2+^ ions were recorded in the case of the most porous systems (D3, D4, K2), which can be associated with their increased adsorption surface (D3-VTC – 61 μg; D4-VTC – 67 μg; K2-VTC – 60 μg per 1 mg of the system). Data obtained for materials of similar composition but lower porosity—e.g., L2-VTC concerning K2-VTC—additionally confirm the influence of the porous structure on the amount of introduced/released calcium. The lowest amounts of Ca^2+^ available for quantification within 30 min were observed for the least porous of the tested systems—E4 and F4 (36 μg per 1 mg of the system). Several overlapping factors may be responsible for such findings: smaller adsorption surface resulting from limited porosity, difficult ion diffusion inside the denser matrix structure, as well as the possibility of strong interactions of Ca^2+^ ions with numerous carboxyl groups (−COOH), which limits their release under the applied measurement conditions.

#### 3.4.3 EE of TMZ-βCD Examination

For all tested materials, the determined content of TMZ-βCD complexes (Figure 5A) was similar, and TMZ encapsulation efficiency totaled values within the range of around 44–50%. Regardless of the systems’ porosity parameters, almost no significant differences were observed in this parameter (except for D3-VTC vs K2-VTC). Each flake-like material could absorb the TMZ in the form of surface-hydrophilic, inclusive complexes to a comparable extent. This proves that materials’ architecture and chemical composition enable interactions with TMZ-βCD complexes and their further effective encapsulation. The only significant difference in TMZ EE was noted between D3-VTC (50%) and K2-VTC (43.8%), two materials with high porosity. It seems that the higher share of open pores (3.1 cm^3^/g for K2 vs 2.3 cm^3^/g for D3) and the looser biopolymeric network favor an easy diffusion of the complexes during the encapsulation stage and may also foster their reverse diffusion, simultaneously reducing TMZ EE in K2-VTC. This theory, concerning more facile medium inflow to the K2 matrix interior, can be indirectly confirmed based on swelling results, where the higher SD was observed for K2–274% compared to D3–217%.

**Figure 5:**
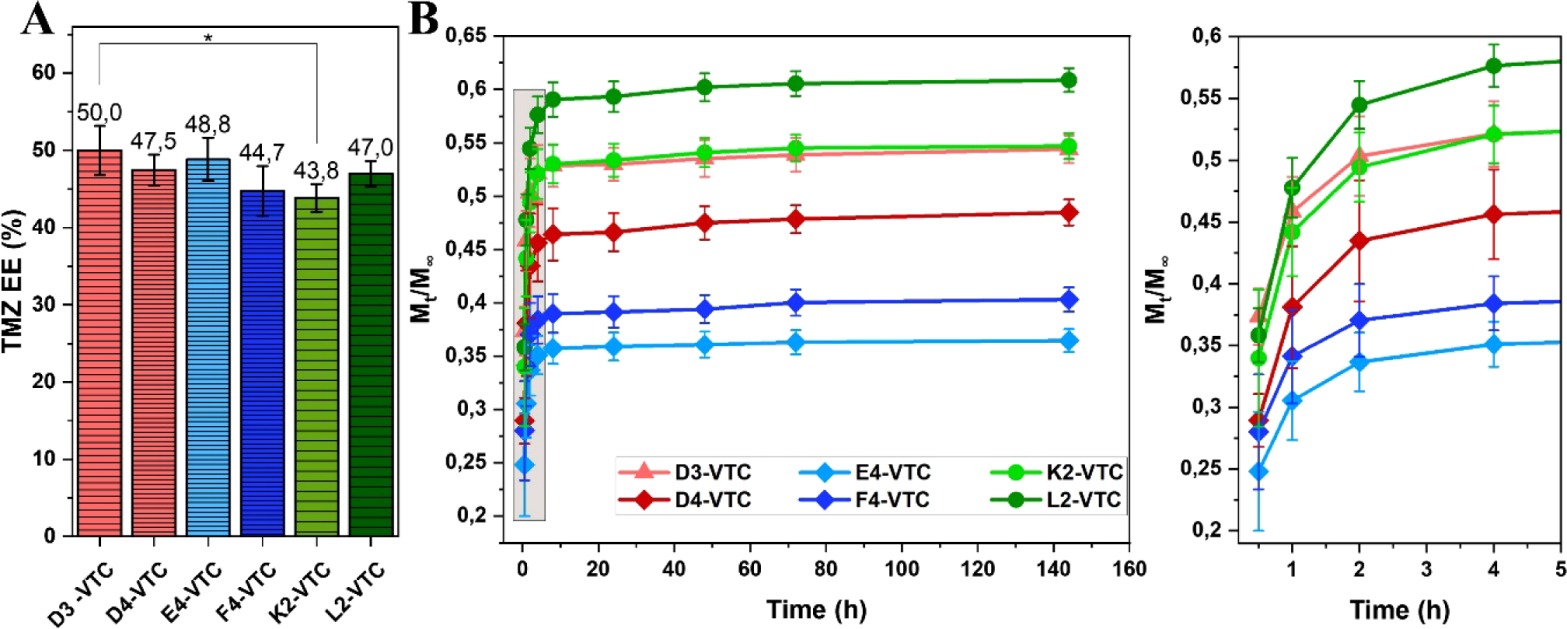
Encapsulation efficiency of TMZ in flake-like materials (A), and drug release profiles from the corresponding systems (B).

#### 3.4.4 In Vitro Release Study of TMZ

In developed systems, the inclusion complexes are likely stabilized within the matrix by numerous hydrogen bonds that may occur between the −OH groups of the cyclodextrin^55^ (21 −OH groups per cyclodextrin molecule) and the functional side groups (−COOH, −NH_2_, −OH) present in the densely cross-linked biopolymer chains forming a three-dimensional network. Therefore, it appears more likely that the drug itself is released rather than the entire complex, as the latter may be stabilized through multiple hydrogen bonds simultaneously. Furthermore, once TMZ leaves the βCD, it can undergo more facile diffusion through the matrix, out of the material, than the larger complex, especially due to the mobility of the TMZ-βCD, which is able to continuously form hydrogen bonds that would slow its release. While the complex moves within the material, TMZ has a chance to be liberated at any point along its path.

Furthermore, the hydrolysis of TMZ and the formation of MTIC alter the compound’s affinity for β-cyclodextrin. Studies on the interactions of TMZ and MTIC with model biological membranes have shown that MTIC exhibited significantly lower affinity for them, which results from its lower lipophilicity than TMZ.^56^ The change in the lipophilicity of MTIC molecules suggests that they do not perform inclusion into nonpolar cyclodextrin cavities as readily as TMZ, especially under dilute conditions.^57,58^ Therefore, it is presumed that if the complex is released and TMZ is degraded, then the resulting products no longer possess the same ability to form stable complexes with βCD under experimental conditions and remain in free form.

The temozolomide release profile was tested in simulated physiological conditions (pH 7.4, 37 °C) for six materials with varying biopolymeric matrix weight ratios (D3-VTC, D4-VTC, E4-VTC, F4-VTC, K2-VTC, L2-VTC). The gathered results are presented in Figure 5B, showing the fraction of drug released, defined as *M_t_*/*M*_∞_ (the compound released from the material at a given time/total amount of the drug), relative to the function of time. As observed, the temozolomide introduced into the materials in the inclusive complexes (TMZ-βCD) was released at different levels for all tested systems.

The lowest relative amounts of the released TMZ after 144 h were detected for materials with a hydrogel network composed of 50% HA_mod_ (0.36–E4-VTC, 0.40–F4-VTC) based on biopolymeric matrices characterized by the lowest porosity (E4–59.04%, F4–46.33%) as well as the lowest total pore volume (E4–0.519 cm^3^/g, F4–0.443 cm^3^/g) among all hydrogel networks. Interestingly, higher amounts of the drug were released from the less porous F4-based material (F4-VTC) than from the E4-based material (E4-VTC). The probable explanation of this phenomenon is the deeper complexes’ penetration into the E4 matrix than F4. In the latter, due to more difficult permeation through fewer open pores, a large part of the TMZ-βCD complexes was localized near the flake’s surface, where the diffusion occurs rapidly during the release experiment. A similar effect regarding normalized drug release after 144 h seems to be observed between K2-VTC (0.55) and L2-VTC (0.61), where TMZ-βCD complexes were rather absorbed close to the edge of the material as a result of the lower porosity parameters of L2.

Additionally, the comparison between extreme release profiles for systems developed on biopolymeric networks with lower open pore volumes than the rest (E4–0.519, F4–0.443, L2–0.757 cm^3^/g) demonstrated that interactions within the hydrogel matrix play a crucial role in drug diffusion during the release experiment. Due to their higher content of hydrophilic groups, the two former systems containing 50% hyaluronic acid (E4-VTC and F4-VTC) allowed for deeper drug incorporation during the loading process and a more gradual release. In contrast, in the 50% chitosan-based system (L2-VTC), the drug complexes were mainly introduced into the top layers of the material. This was likely caused by hindered diffusion, resulting from a lower level of hydrophilic groups, which led to a more surface-oriented release profile.

In the case of gelatin-based systems (D3-VTC and D4-VTC), the influence of the genipin concentration (4.64 mM for D3-VTC, 6.63 mM for D4-VTC) can be discussed. Considering the same matrix composition, we can conclude that higher cross-linking agent content could increase cross-linking density (observed indirectly by swelling degree) and seal the structure. Thereby, the TMZ burst release of D4-VTC was reduced due to impeded drug diffusion by a tighter matrix network compared to D3-VTC.

From the observations, it can be inferred that three main parameters, such as cross-linking agent amount, porosity, and internal interactions, influenced the release profile of the fabricated composite materials based on a biopolymeric matrix.

#### 3.4.5 Release Kinetics

Describing release kinetics from flake-like composites based on the biopolymeric matrix is challenging since the material behavior is complex and significantly affects the release profile. We attempted to fit experimental data to commonly applied kinetic models, such as Higuchi and Korsmeyer-Peppas, to gain insight into the mechanisms governing drug release kinetics. However, their correlation was poor.

The applied first-order kinetic model (Figure S3) demonstrated a good fit for the experimental data (Table 5). The calculated rate constants (k) for all systems indicated a tendency toward the rapid initial release of TMZ at the beginning of the experiment.

**Table 5:** Parameters of Mathematical Models Fitted to Experimental Data Obtained for Six Materials with TMZ

		Material					
Model	Parameters	D3-VTC	D4-VTC	E4-VTC	F4- VTC	K2-VTC	L2-VTC
**First order**	***R*** ^**2**^	0.9532	0.9740	0.8681	0.8221	0.8787	0.9506
	***F***_**max**_	0.5341	0.4757	0.3600	0.3955	0.5390	0.5973
	**k**	2.2764	1.8304	1.9760	2.1833	1.6994	1.6775
Weibull 3	***R*** ^**2**^	0.9839	0.9844	0.9746	0.9263	0.9580	0.9861
	***F***_**max**_	0.5364	0.4771	0.3617	0.3976	0.5417	0.6017
	***a***	0.5447	0.6825	0.5605	0.5490	0.6260	0.6794
	***b***	0.6046	0.6336	0.5341	0.4784	0.5493	0.6484
**Double exponential**	***R*** ^**2**^	0.9879	0.9975	0.9531	0.9694	0.9803	0.9922
	**A1**	0.5129	0.4539	0.3513	0.3815	0.5146	0.5546
	**k1**	2.5100	2.001	2.1640	2.4658	1.9818	1.9916
	**A2**	0.0292	0.0321	0.0140	0.0225	0.0320	0.0505
	**k2**	0.0403	0.0214	0.0221	0.0221	0.0434	0.1049

A better fit was obtained using the Weibull-3 model (Figure S4), which accounts for a more complex release mechanism. The estimated *F*_max_ values corresponded well with the actual experimental results (Table 5). At the same time, the shape (b) and scale (a) parameters confirmed a burst release occurring at early time points in all cases. The lowest values of both a and b for HA_mod_-based systems (E4-VTC, F4-VTC) reflected the most substantial burst release effect among all systems. A comparison of shape and scale parameters for D3-VTC and D4-VTC indicated that higher cross-linking agent content weakened the effect. Similarly, the less porous structure of L2 compared to K2 also influenced a reduced burst release.

Furthermore, the best overall fit for almost all systems was achieved using the double-exponential model (Figure S5), which captures two-phase release kinetics. The parameters A1 and A2 represent the drug fractions released during the fast (burst) and slower phases, respectively, while parameters k1 and k2 describe their associated release rates (Table 5). Based on the A1 values, the highest amount of the drug was released during the first phase for chitosan-based materials (K2-VTC ∼ 51%, L2-VTC ∼ 55%) and a looser system with a gelatin-dominant share (D3-VTC ∼ 51%). A significantly reduced amount of the drug that underwent rapid release at the beginning was observed for flakes with a matrix composed mainly of HA_mod_ (E4-VTC ∼ 35%, F4-VTC ∼ 38%) and D4-VTC ∼ 45%. Low values of A2 and k2 indicated that a second stage with a slow drug release occurs for all tested formulations.

Based on the behavior of our flake-like biopolymeric materials, the release of TMZ initiates with a pronounced burst phase, driven by the rapid diffusion of drug complexes localized near the material’s surface. Subsequently, the flakes undergo swelling, during which the polymeric network is densified, entrapping the remaining drug complexes within the internal matrix. The structural transition effectively creates a physical diffusion barrier and slows the further release. As a result, the initial rapid outflow is followed by a sustained and markedly limited release phase, indicating strong matrix–drug interactions and hindered transport through the swollen hydrogel structure.

The extent of burst release appears to be strongly correlated with the drug’s ability to penetrate the system during the loading stage—the easier the transport into the inner structure, the fewer complexes remain near the surface and edges. Accordingly, materials based predominantly on chitosan retained the largest amount of the drug at the surface. In contrast, gelatin-based systems allowed for deeper drug penetration, and systems mainly containing HA_mod_ exhibited the lowest surface retention, suggesting the most effective incorporation of the drug into the matrix.

### 3.5 Antibacterial Activity In Vitro

Vancomycin is an antibiotic that is effective against Gram-positive bacteria and is recommended for patients after neurosurgical treatment;^22,24^ thus, it was chosen to be incorporated into our materials to give them antibacterial potential. Moreover, local administration of vancomycin is strongly preferred, as it reduces hospitalization time, minimizes systemic side effects, and significantly improves the patient’s quality of life.^24^ The antibacterial activity of the obtained composites was tested at the same *Staphylococcus aureus* concentration using commercially available hemostatic materials (TachoSil, Clinisponge, Reoxcel) as controls.

The zone of inhibition (Figure 6A–C) for all the flake-like materials reached 18.4–24.8 mm and was higher than for Reoxcel (17.5 mm), the only commercial system exhibiting an antibacterial effect (for statistical analysis, see Figure S6). A bigger inhibition diameter was noted for D3-VTC than D4-VTC (with matrices composed of 50% gelatin), even though the vancomycin encapsulation efficiency (VANC EE) was approximately 14% higher for the latter system. A comparison of those results implies that VANC encapsulated in the chitosan particles embedded in a matrix fabricated with lower cross-linking agent concentrations exhibited better availability for acting against bacteria. The most significant differences in the inhibition zone diameters were observed for the systems in which hyaluronic acid was the dominant matrix component (18.4 mm – E4-VTC; 23.1 mm – F4-VTC). Although both systems exhibited similarly high vancomycin encapsulation efficiency (VANC EE: 84.4% for E4-VTC and 82.1% for F4-VTC) and low porosity values, their antibacterial performance differed significantly. These differences are most likely attributed to variations in composition. The higher chitosan content in E4-VTC compared to F4-VTC may have resulted in a denser structure that limited antibiotic release, thereby reducing antibacterial activity. A similar trend was observed in the TMZ release profiles (Section 3.4.4). The most effective inhibition against *Staphylococcus aureus* was observed for systems based on matrices with a higher proportion of chitosan (24.8 mm – K2-VTC; 24.0 mm – L2-VTC). In this case, the theory of antibiotic availability also seems to be applicable. Although both systems contained the same amount of chitosan and were cross-linked with the same genipin concentration, L2-VTC exhibited slightly higher vancomycin encapsulation efficiency (79.3%) than K2-VTC (76.4%), yet demonstrated a comparable inhibition zone. This difference may be explained by the higher porosity of K2-VTC, which likely facilitated a more efficient antibiotic release, resulting in enhanced antibacterial activity.

**Figure 6:**
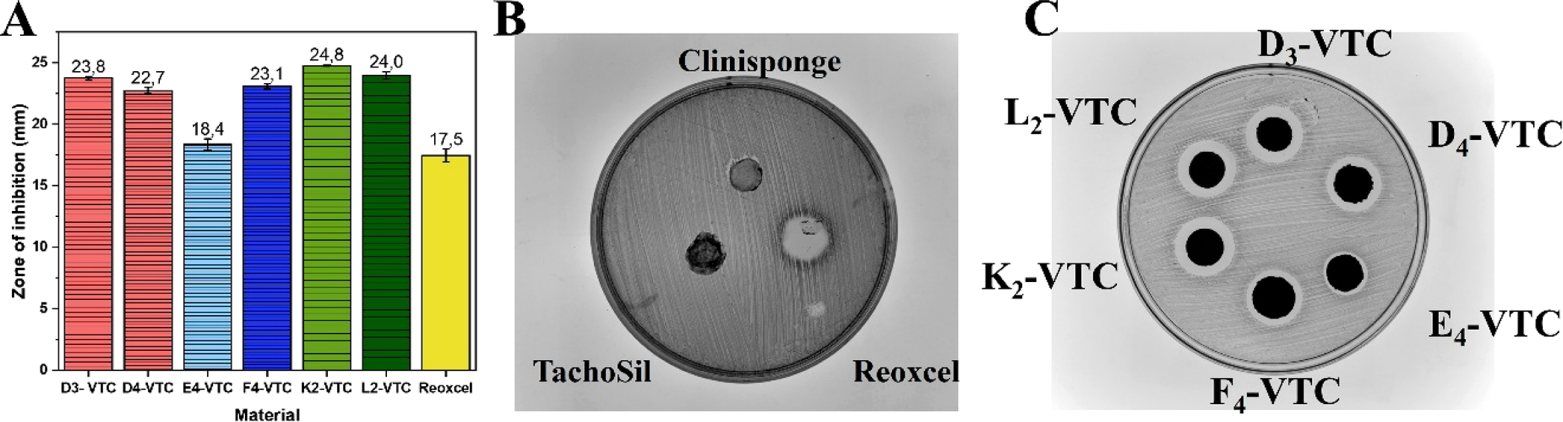
Antibacterial effect against *Staphylococcus aureus* evaluated based on the size of the inhibition zones (A), with corresponding representative images of commercially available hemostatic materials (B) and composite flake-like systems (C).

The results indicate that both the concentration of the antibiotic and its accessibility, influenced by the composition, porosity, and structural arrangement of the material, are key factors affecting materials’ antibacterial effectiveness.

### 3.6 Hydrogel Flakes with Temozolomide Have Strong Anti-Cancer Activity and Are Biocompatible

To analyze the anticancer activity of the obtained materials with TMZ, we conducted experiments using U-251 MG glioma cells directly exposed to the materials placed on the cell layers (Figure 7A). As shown in Figure 7B, after 72 h, we observed only a slight decrease in the viability of glioma cells exposed to all types of tested materials. However, after 7 days of exposure, the viability of glioma cells significantly decreased for all materials containing TMZ, dropping to 40% compared to the control cells that were unexposed to the tested materials. This indicates efficient release and high biological activity of TMZ. In contrast, the viability of glioma cells incubated with materials that did not contain TMZ decreased to 80% (compared to untreated cells), suggesting low toxicity for those materials. Given that all six formulations exhibited properties implying their suitability for the intended application, for further studies, we selected materials differing in the major constituent of the matrix, containing 50% gelatin, lysine-modified hyaluronic acid, or chitosan—D4-VTC, F4-VTC, and K2-VTC, respectively. As those systems revealed some differences in physicochemical characteristics but simultaneously showed antibacterial performance, we aimed to assess their composition’s influence on biological features. Since TMZ is a potent DNA damage-inducing agent, it is expected to strongly impact cell proliferation by inhibiting cell cycle progression.^59,60^ As shown in Figure 7C, significant changes in the distribution of glioma cells across specific cell cycle phases were observed after 72 h of exposure to all materials containing TMZ. Notably, there was a strong decrease in the population of cells in the G1 phase. In contrast, we observed an accumulation of cells in the S/G2/M phases, indicating perturbations in DNA replication and mitosis due to TMZ-induced DNA damage. These cells could not complete the cell cycle and progress to the G1 phase, which explains the substantial reduction in the G1 population. Our results suggest that the tested materials slowly release TMZ, accumulating in the medium and retaining its biological activity. This primarily manifests through inhibiting glioma cell proliferation and, ultimately, decreasing the viability of glioma cells due to the accumulation of DNA damage.

**Figure 7:**
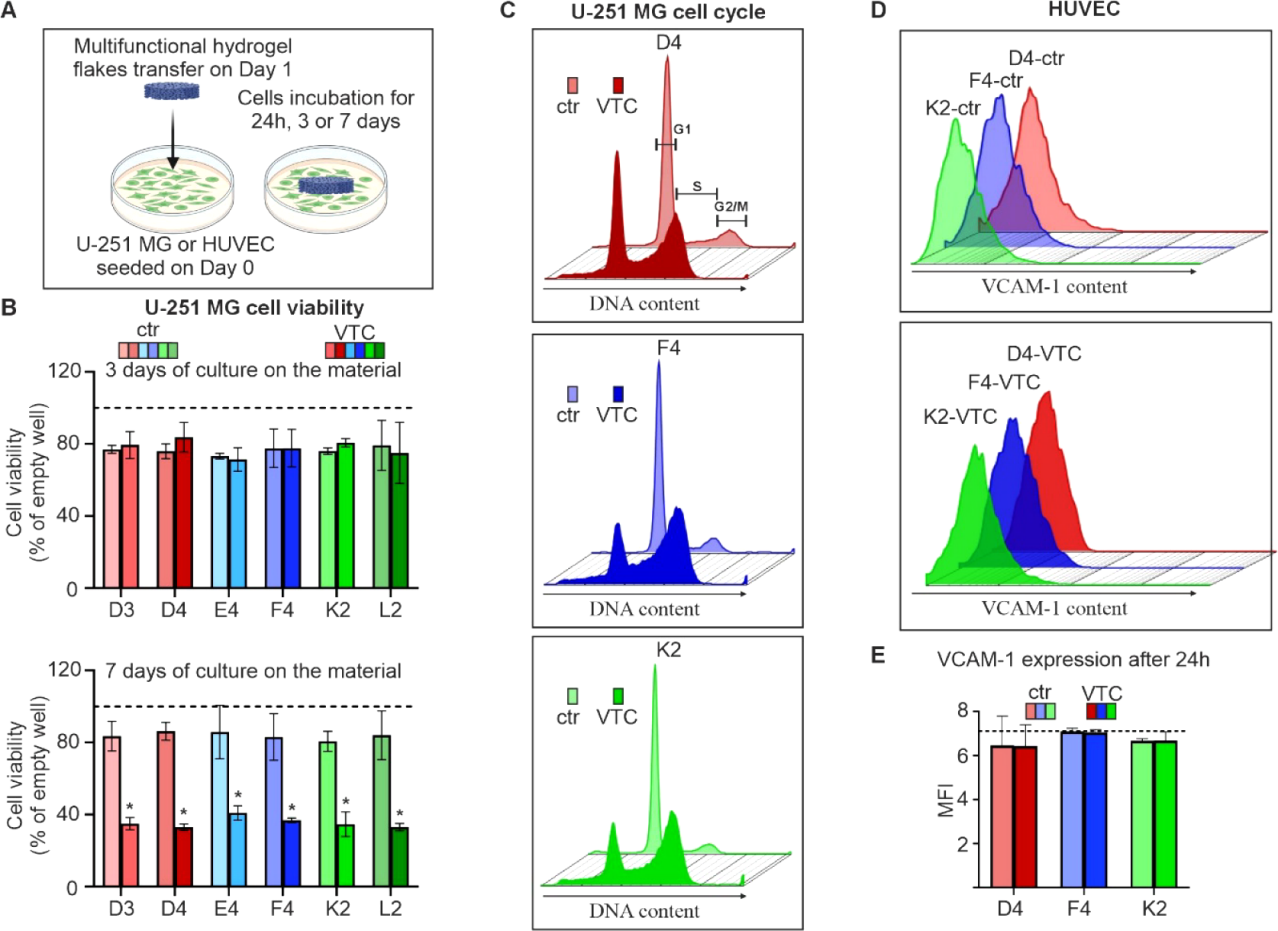
Interaction of the materials with human cells. (A) Schematic representation of direct incubation of cells with the hydrogel flakes. (B) Viability of U-251 MG cells exposed to materials for 72 h or 7 days. (C) The effect of selected materials on cell cycle progression performed after 24-h incubation using flow cytometry with propidium iodide (PI) staining after cell fixation. (D) Histograms of analysis of the proinflammatory activity of materials against HUVEC cells by VCAM-1 detection using a flow cytometer and (E) summarized mean fluorescence intensity (MFI). The gating strategy for flow cytometry analysis is demonstrated in Figure S1.

Having confirmed the anticancer activity, further studies were focused on biocompatibility analyses. These analyses involved human primary endothelial cells (HUVECs) since we assumed that after the therapeutic materials are placed in the patient’s brain after intraoperative tumor removal, they could come into contact with blood vessels and might affect the function of endothelial cells. The layers of HUVECs were exposed to the tested materials placed above the cells for 24 h. Then, we analyzed the expression of VCAM-1 in the HUVECs’ membrane, which indicates a proinflammatory response induced in the endothelium. As demonstrated in Figure 7D,E, none of the tested materials induced the expression of VCAM-1. At the same time, the proper response of the cells to proinflammatory factors was confirmed using the TNF (Figure S1B). Finally, we analyzed the cytotoxicity of the compounds released from the tested materials. For these analyses, we chose cells isolated from human blood (red cells and PBMCs) and HepG2 cells, which are a model of human hepatocytes. We postulate that these cells would be particularly exposed and vulnerable to potentially toxic molecules released from the materials during their application *in vivo*. The cells were exposed to the extracts collected from the materials (as described in Section 2), and then the extracts’ hemolytic and cytotoxic activity was analyzed. According to Figure S7, we observed only a slight decrease in PBMC viability when exposed to some D4, K2, D4-VTC, and K2-VTC extracts.

### 3.7 Analysis of Hemostatic Properties of Tested Materials

We used freshly isolated human peripheral blood, which was recalcified before the experiments to evaluate the hemostatic properties of the tested materials. According to the manufacturers, the hemostatic effect of commercially available Reoxcel^61^ and Clinisponge^62^ results primarily from their physical structure, which, after swelling, acts as a barrier and facilitates platelet accumulation, thereby supporting physiological hemostasis. In contrast, TachoSil^63^ additionally exhibits an active mechanism of action due to the presence of thrombin and fibrinogen. Upon contact with blood, these components are activated and initiate the final step of the coagulation cascade, resulting in fibrinogen-to-fibrin conversion. The fibrin fibers form a clot that adheres to the collagen sponge, achieving hemostasis and providing an effective seal. Our hydrogel flakes contain two components that make them promising candidates for hemostatic applications: (1) chitosan and (2) calcium ions. The hemostatic activity of chitosan results primarily from its high resorption ability, leading to blood cell concentration and facilitated clot formation.^64^ Therefore, we examined how well the tested materials absorbed blood by transferring 0.5 mL of blood onto the materials placed in the wells of six-well plates (Figure 8A). As demonstrated in Figure 8B and Movie S1, all of our tested materials exhibited efficient blood absorption, soaking up the blood immediately upon application. In contrast, the control materials—Reoxcel, TachoSil, and Clinisponge—showed significantly lower absorption properties. Instead of being absorbed, the blood flowed off their surfaces into the well, as shown in Figure 8B and Movie S2. After a 15 min incubation period, we gently rinsed the materials with distilled water to remove untrapped or unclotted cells. The fluids containing blood cells unbound within the materials were collected in fresh tubes. Following cell lysis, we measured the released hemoglobin and compared the percentage of nonclotted cells (see Figure 8A). Figure 8C shows that the percentage of cells washed out from all of our materials was very low, indicating that nearly all cells were trapped, bound, or clotted within the materials after the blood transfer. Notably, K2-VTC (calcium-ion-containing materials) activated clotting more efficiently than K2. The hemostatic properties of D4 and F4 were comparable to those of their calcium-ion-enhanced versions. Interestingly, for TachoSil, we observed a higher percentage of unclotted cells, which can be attributed to the lower material’s absorption properties.

**Figure 8:**
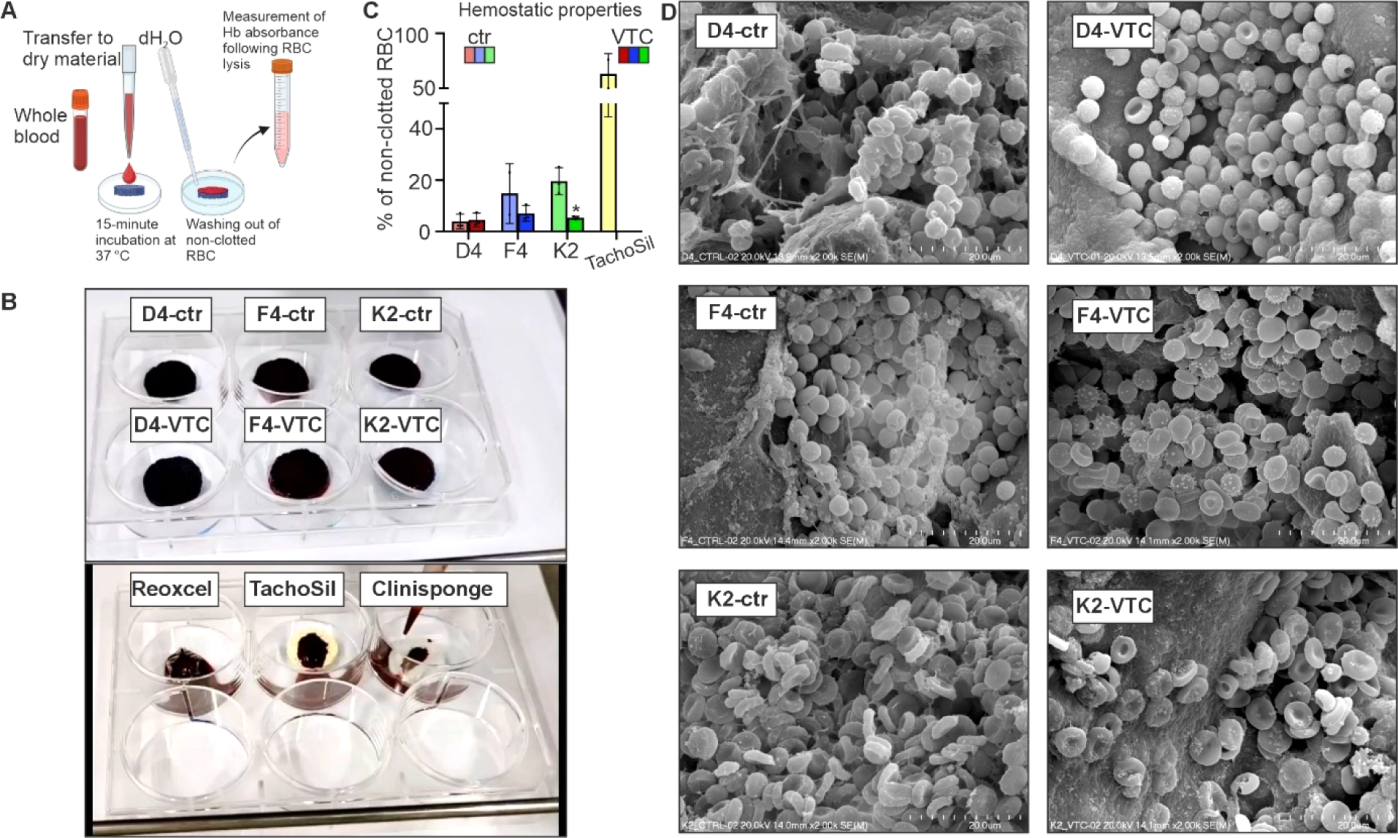
Hemostatic properties of materials. (A) Schematic presentation of experiments verifying absorbing and hemostatic properties of tested materials. (B) Pictures presenting absorption properties. (C) Comparison of the percentage of free cells (unclotted) that remained in the wells after transferring the blood into tested materials. This analysis was performed based on the level of hemoglobin released from the cells collected from the wells after incubating the blood with materials. (D) SEM images demonstrating clot formation inside the tested materials.

The positively charged amino groups in chitosan interact with the negatively charged cell membranes critical for clotting cells, such as red blood cells and platelets.^64–66^ This interaction is essential for hemostasis during bleeding, as it promotes cell activation, adhesion, morphological changes, and aggregation.^67^ These processes collectively lead to clot formation, occurring independently of the plasma coagulation cascade. Additionally, calcium ions in the surrounding environment may enhance this response by improving the interaction between cells and chitosan. We used scanning electron microscopy (SEM) to examine the clotting process within the tested materials. As shown in Figure 8D, we observed the presence of platelet and red blood cell aggregates, red cells exhibiting altered morphology (echinocytes), and tightly packed polyhedral erythrocytes, all characteristic indicators of clot formation.^68^ In our opinion, the effect of calcium ion addition seems visible predominantly for K2-VTC materials. In this case, most SEM images demonstrated the presence of a clot composed of red cells and platelet aggregates with fibrin or polyhedrocytes (Figures 8D and S8). During clot contraction, activated platelets exert contractile forces on the fibrin network, leading to the compression of erythrocytes in the clot core. As a result, red blood cells undergo a morphological transformation from their native biconcave disc shape into tightly packed polyhedral structures, known as polyhedrocytes. This transformation enables a highly compact cellular architecture, creating an almost impermeable barrier that prevents further bleeding. The presence of polyhedrocytes indicates efficient clot contraction and is associated with increased clot mechanical stability and resistance to fibrinolysis.^69^

## 4 Conclusion

The multifunctional hydrogel-based flakes designed to address the issues of local GBL therapy, bacterial neuroinfections, and the bleeding control needed during tumor resection are presented within this study. The developed materials comprise TMZ and vancomycin, loaded into cyclodextrin/polymeric capsules and embedded into gelatin/hyaluronic acid/chitosan-based hydrogel films cross-linked with genipin. The calcium cations were also incorporated into the systems to enhance the hemostatic properties. That designed formulation was subsequently freeze-dried to serve in flake-like forms, enabling the lining of the surgery site during surgical resection, transferring both TMZ and VAN directly into the surrounding brain parenchyma (the most common site of recurrence). Such a route of drug delivery represents unique features since systemic side effects are minimized while the drug dose is increased. The developed systems were characterized by their designed functionalities. Therefore, biological evaluation *in vitro/ex vivo*, antibacterial activity tests *in vitro*, drug release study in various conditions, and physicochemical feature assessments were performed. We have found that the systems of various features (swelling, degradation profile, porosity) can be designed by playing with hydrogel composition and cross-linking degree. Importantly, all tested materials exhibited high stability, indicating their favorable potential for gradual degradation and control of drug release. The revealed similarity of the porosity and pore sizes within the obtained biopolymeric matrices to the commercial hemostatic systems (Reoxcel, TachoSil, Clinisponge) would ensure the flow of platelets as well as erythrocytes, which are necessary for the clotting process. Depending on the matrix composition, the ability of TMZ-cyclodextrin complexes to penetrate into the systems at the loading stage varies significantly, which affects the drug release profile. We have also verified the antibacterial activity of the resulting systems, showing that the antibiotic concentration and the matrix’s physicochemical properties are key factors influencing this functionality. Since all tested formulations (with TMZ, VANC, and Ca^2+^ loaded) exhibited properties implying their suitability for the intended application for biological evaluation *in vitro/ex vivo*, we have selected materials differing in the major constituent of the matrix: D4-VTC, F4-VTC, and K2-VTC, respectively.

Our investigations on human glioma cells, which included assessments of cell viability and cell cycle progression, confirmed the anticancer activity of TMZ when incorporated in the tested materials. Furthermore, we established that these materials exhibit a high biocompatibility by analyzing their interactions with normal human cells. Lastly, we observed remarkable blood absorption properties of the tested materials and their hemostatic activity, as evidenced by clot formation within the materials, as confirmed through SEM imaging.

Our findings confirmed that the resulting multifunctional long-acting delivery system allows for sustained TMZ release and possesses antimicrobial properties while simultaneously displaying the hemostatic potential. Overall, the work presented here introduces an innovative and deeply thought-out approach to the more effective and safer use of temozolomide in treating glioma. The unique clinical conditions related to the local CNS drug administration, providing each of them with dedicated solutions, were analyzed within this study. The presented methods and results describe the invention and its interactions with the environment, which is an important step toward clinical application. However, an *in vivo* biocompatibility and therapeutic efficacy study should be considered as the next step in verifying/proving the implantable potential of the developed systems.

## Supplementary Material







## Data Availability

The datasets generated and/or analyzed during the current study are available in the RODBUK repository: https://doi:10.57903/UJ/ZOVXYB.

## Notes

Movie presenting the developed materials’ blood absorption (Movie S1) (MP4)
Movie presenting the commercial materials’ blood absorption (MP4)
Gating strategy. Cell cycle analysis and VCAM-1 measurement (Figure S1); degradation study results with statistical analysis using the Student’s *t*-test (Figure S2); first-order model fit (Figure S3); Weibull 3 model fit (Figure S4); double exponential model fit (Figure S5); antibacterial activity against *Staphylococcus aureus*, with statistical evaluation performed using Student’s *t-*test (Figure S6); biocompatibility analysis using extracts collected from materials at various time points and containing the potentially toxic compounds released from the materials (Figure S1); SEM images demonstrating clot formation inside the tested materials (Figurer S2) (PDF)

## References

[1] Krajcer A., Grzywna E., Lewandowska-Łańcucka J. (2023). Strategies
Increasing the Effectiveness of Temozolomide at Various Levels of
Anti-GBL Therapy. Biomed. Pharmacother..

[2] Wesseling P., Capper D. (2018). WHO 2016 Classification
of Gliomas. Neuropathol. Appl. Neurobiol..

[3] Anjum K., Shagufta B. I., Abbas S. Q., Patel S., Khan I., Shah S. A. A., Akhter N., Hassan S. S. (2017). ul. Current Status
and Future Therapeutic Perspectives of Glioblastoma Multiforme (GBM)
Therapy: A Review. Biomed. Pharmacother..

[4] Tan A. C., Ashley D. M., López G. Y., Malinzak M., Friedman H. S., Khasraw M. (2020). Management of Glioblastoma:
State of the Art and Future
Directions. Ca-Cancer J. Clin..

[5] Ge Y., Lu H., Martí J. (2025). Influence
of Local Ordering in the Permeation of Temozolomide
through the Brain Plasmatic Membrane. Biophys.
Chem..

[6] He Q., Liu J., Liang J., Liu X., Li W., Liu Z., Ding Z., Tuo D. (2018). Towards Improvements for Penetrating
the Blood–Brain Barrier—Recent Progress from a Material
and Pharmaceutical Perspective. Cells.

[7] Krajcer A., Hinz A., Bzowska M., Stankiewicz S., Słomka J., Horak W., Grzywna E., Lewandowska-Łańcucka J. (2025). Hydrogel-Based
Implantable System for Local Delivery of Temozolomide in Postsurgical
Brain Cancer Therapy. Chem. Eng. J..

[8] Parkins C. C., McAbee J. H., Ruff L., Wendler A., Mair R., Gilbertson R. J., Watts C., Scherman O. A. (2021). Mechanically
Matching
the Rheological Properties of Brain Tissue for Drug-Delivery in Human
Glioblastoma Models. Biomaterials.

[9] Shapira-Furman T., Serra R., Gorelick N., Doglioli M., Tagliaferri V., Cecia A., Peters M., Kumar A., Rottenberg Y., Langer R., Brem H., Tyler B., Domb A. J. (2019). Biodegradable
Wafers Releasing Temozolomide and Carmustine for the Treatment of
Brain Cancer. J. Controlled Release.

[10] Chowdhary S. A., Ryken T., Newton H. B. (2015). Survival
Outcomes and Safety of Carmustine
Wafers in the Treatment of High-Grade Gliomas: A Meta-Analysis. J. Neurooncol..

[11] Sage W., Guilfoyle M., Luney C., Young A., Sinha R., Sgubin D., McAbee J. H., Ma R., Jefferies S., Jena R., Harris F., Allinson K., Matys T., Qian W., Santarius T., Price S., Watts C. (2018). Local Alkylating
Chemotherapy Applied Immediately after 5-ALA Guided Resection of Glioblastoma
Does Not Provide Additional Benefit. J. Neurooncol..

[12] Jiang B. P., Zhang L., Zhu Y., Shen X. C., Ji S. C., Tan X. Y., Cheng L., Liang H. (2015). Water-Soluble Hyaluronic
Acid-Hybridized Polyaniline Nanoparticles for Effectively Targeted
Photothermal Therapy. J. Mater. Chem. B.

[13] Klara J., Onak S., Kowalczyk A., Wójcik K., Lewandowska-Łańcucka J. (2024). Photocrosslinked
Gelatin/Chondroitin
Sulfate/Chitosan-Based Composites with Tunable Multifunctionality
for Bone Tissue Regeneration. Int. J. Biol.
Macromol..

[14] Aurand E. R., Wagner J., Lanning C., Bjugstad K. B. (2012). Building Biocompatible
Hydrogels for Tissue Engineering of the Brain and Spinal Cord. J. Funct. Biomater..

[15] Silant’ev V. E., Belousov A. S., Trukhin F. O., Struppul N. E., Shmelev M. E., Patlay A. A., Shatilov R. A., Kumeiko V. V. (2024). Rational Design
of Pectin–Chitosan Polyelectrolyte Nanoparticles for Enhanced
Temozolomide Delivery in Brain Tumor Therapy. Biomedicines.

[16] Kasapidou P. M., de Montullé E. L., Dembélé K. P., Mutel A., Desrues L., Gubala V., Castel H. (2021). Hyaluronic
Acid-Based Hydrogels Loaded with Chemoattractant and Anticancer Drug
- New Formulation for Attracting and Tackling Glioma Cells. Soft Matter..

[17] Yang L., Gao S., Asghar S., Liu G., Song J., Wang X., Ping Q., Zhang C., Xiao Y. (2015). Hyaluronic Acid/Chitosan
Nanoparticles for Delivery of Curcuminoid and Its in Vitro Evaluation
in Glioma Cells. Int. J. Biol. Macromol..

[18] Lee J. H., Kim H. W. (2018). Emerging Properties
of Hydrogels in Tissue Engineering. J. Tissue
Eng..

[19] Muzzarelli R. A. A. (2009). Genipin-Crosslinked
Chitosan Hydrogels as Biomedical and Pharmaceutical Aids. Carbohydr. Polym..

[20] MacAya D., Ng K. K., Spector M. (2011). Injectable
Collagen-Genipin Gel for
the Treatment of Spinal Cord Injury: In Vitro Studies. Adv. Funct. Mater..

[21] Moorthy R. K., Rajshekhar V. (2008). Management
of Brain Abscess: An Overview. Neurosurg. Focus.

[22] Davani F., Alishahi M., Sabzi M., Khorram M., Arastehfar A., Zomorodian K. (2021). Dual Drug
Delivery of Vancomycin and Imipenem/Cilastatin
by Coaxial Nanofibers for Treatment of Diabetic Foot Ulcer Infections. Mater. Sci. Eng., C.

[23] Bruniera F. R., Ferreira F. M., Saviolli L. R. M., Bacci M. R., Feder D., Pedreira M. D. L. G., Peterlini M. A. S., Azzalis L. A., Junqueira V. B. C., Fonseca F. L. A. (2015). The Use of Vancomycin with Its Therapeutic
and Adverse Effects: A Review. Eur. Rev. Med.
Pharmacol. Sci..

[24] Tseng Y. Y., Kao Y. C., Liao J. Y., Chen W. A., Liu S. J. (2013). Biodegradable
Drug-Eluting Poly­[Lactic-Co-Glycol Acid] Nanofibers for the Sustainable
Delivery of Vancomycin to Brain Tissue: In Vitro and in Vivo Studies. ACS Chem. Neurosci..

[25] Florczyk A., Krajcer A., Wójcik K., Lewandowska-Łańcucka J. (2024). Innovative
Vancomycin-Loaded Hydrogel-Based Systems – New Opportunities
for the Antibiotic Therapy. Int. J. Nanomed..

[26] Ding C., Tian M., Feng R., Dang Y., Zhang M. (2020). Novel Self-Healing
Hydrogel with Injectable, PH-Responsive, Strain-Sensitive, Promoting
Wound-Healing, and Hemostatic Properties Based on Collagen and Chitosan. ACS Biomater. Sci. Eng..

[27] Muthiah
Pillai N. S., Eswar K., Amirthalingam S., Mony U., Kerala Varma P., Jayakumar R. (2019). Injectable
Nano Whitlockite Incorporated Chitosan Hydrogel for Effective Hemostasis. ACS Appl. Bio Mater..

[28] Sundaram M. N., Mony U., Varma P. K., Jayakumar R. (2021). Vasoconstrictor
and Coagulation Activator Entrapped Chitosan Based Composite Hydrogel
for Rapid Bleeding Control. Carbohydr. Polym..

[29] Kristiansen G. K., Andersen M. D. (2011). Reversible Activation of Cellular
Factor XIII by Calcium. J. Biol. Chem..

[30] Gilarska A., Lewandowska-Łańcucka J., Guzdek-Zając K., Karewicz A., Horak W., Lach R., Wójcik K., Nowakowska M. (2020). Bioactive yet Antimicrobial Structurally Stable Collagen/Chitosan/Lysine
Functionalized Hyaluronic Acid – Based Injectable Hydrogels
for Potential Bone Tissue Engineering Applications. Int. J. Biol. Macromol..

[31] Gürten B., Yenigül E., Sezer A. D., Malta S. (2018). Complexation and Enhancement
of Temozolomide Solubility with Cyclodextrins. Brazilian J. Pharm. Sci..

[32] Davidenko N., Schuster C. F., Bax D. V., Raynal N., Farndale R. W., Best S. M., Cameron R. E. (2015). Control
of Crosslinking for Tailoring
Collagen-Based Scaffolds Stability and Mechanics. Acta Biomater..

[33] Sharaf
El-Din M. K., Ibrahim F., El-Deen A. K., Shimizu K. (2018). Stability-Indicating
Spectrofluorimetric Method with Enhanced Sensitivity for Determination
of Vancomycin Hydrochloride in Pharmaceuticals and Spiked Human Plasma:
Application to Degradation Kinetics. J. Food
Drug Anal..

[34] Demertzis M. A. (1988). Fluorimetric
Determination of Calcium in Serum with Calcein. Complexation of Calcein
with Calcium and Alkali Metals. Anal. Chim.
Acta.

[35] Zhang Y., Huo M., Zhou J., Zou A., Li W., Yao C., Xie S. (2010). DDSolver: An Add-in Program for Modeling
and Comparison of Drug Dissolution
Profiles. AAPS J..

[36] Follonier N., Doelker E., Cole E. T. (1995). Various
Ways of Modulating the Release
of Diltiazem Hydrochloride from Hot-Melt Extruded Sustained Release
Pellets Prepared Using Polymeric Materials. J. Controlled Release.

[37] Eastoe J. E. (1955). The Amino
Acid Composition of Mammalian Collagen and Gelatin. Biochem. J..

[38] Lewandowska K., Sionkowska A., Grabska-Zielińska S., Michalska-Sionkowska M. (2017). Characterisation
of Chitosan/Hyaluronic Acid Blend Films Modified by Collagen. Prog. Chem. Appl. Chitin Its Deriv..

[39] Butler M. F., Ng Y. F., Pudney P. D. A. (2003). Mechanism and Kinetics of the Crosslinking
Reaction between Biopolymers Containing Primary Amine Groups and Genipin. J. Polym. Sci., Part A: polym. Chem..

[40] Vieira M.G.A., Da Silva M.A., Dos Santos L.O., Beppu M. M. (2011). Natural-Based Plasticizers
and Biopolymer Films: A Review. Eur. Polym.
J..

[41] Bigi A., Cojazzi G., Panzavolta S., Roveri N., Rubini K. (2002). Stabilization
of Gelatin Films by Crosslinking with Genipin. Biomaterials.

[42] Miguel S. P., Ribeiro M. P., Coutinho P. (2021). Biomedical Applications
of Biodegradable
Polymers in Wound Care. Wound Heal. Res. Curr.
Trends Futur. Dir..

[43] Dong Z., Zhao J., Xu J., Deng W., Sun P. (2024). Strongly Adhesive,
Self-Healing, Hemostatic Hydrogel for the Repair of Traumatic Brain
Injury. Biomacromolecules.

[44] Tang-Schomer M. D., White J. D., Tien L. W., Schmitt L. I., Valentin T. M., Graziano D. J., Hopkins A. M., Omenetto F. G., Haydon P. G., Kaplan D. L. (2014). Bioengineered Functional
Brain-like Cortical Tissue. Proc. Natl. Acad.
Sci. U. S. A..

[45] Hudecz D., Khire T., Chung H. L., Adumeau L., Glavin D., Luke E., Nielsen M. S., Dawson K. A., Mcgrath J. L., Yan Y. (2020). Ultrathin Silicon Membranes
For In Situ Optical Analysis Of Nanoparticle
Translocation Across a Human Blood–Brain Barrier Model. ACS Nano.

[46] Handtke S., Thiele T. (2020). Large and Small Platelets —(When) Do They Differ?. J. Thromb. Haemost..

[47] Boroushaki T., Dekamin M. G. (2023). Interactions between
β-Cyclodextrin as a Carrier
for Anti-Cancer Drug Delivery: A Molecular Dynamics Simulation Study. J. Biomol. Struct. Dyn..

[48] Krzak A., Bilewicz R. (2020). Voltammetric/UV–Vis
Study of Temozolomide Inclusion
Complexes with Cyclodextrin Derivatives. Bioelectrochemistry.

[49] Shervington L., Ingham O., Shervington A. (2020). A Novel Series
of Phenolic Temozolomide
(TMZ) Esters with 4 to 5-Fold Increased Potency, Compared to TMZ,
against Glioma Cells Irrespective of MGMT Expression. RSC Adv..

[50] Yuan C., Liu B., Liu H. (2015). Characterization
of Hydroxypropyl-β-Cyclodextrins
with Different Substitution Patterns via FTIR, GC-MS, and TG-DTA. Carbohydr. Polym..

[51] Laszcz M., Kubiszewski M., Jedynak L., Kaczmarska M., Kaczmarek L., Luniewski W., Gabarski K., Witkowska A., Kuziak K., Malińska M. (2013). Identification and Physicochemical
Characteristics of Temozolomide Process-Related Impurities. Molecules.

[52] Liu Y., Weng L., Lin Y., Lin D., Xie L., Zhong T. (2023). Carvacrol/β-Cyclodextrin Inclusion
Complex as a Fumigant to
Control Decay Caused by Penicillium Digitatum on Shatangju Mandarin
Slices. Heliyon.

[53] Ms M., Venkatasubbu G. D. (2024). Modulating
Coagulation via Bioinspired Mesoporous Calcium-
Decorated Silica Nanoparticles for Efficient Fibrin Clot Formation. ACS Appl. Bio Mater..

[54] Granica M., Tymecki Ł. (2019). Analytical Aspects of Smart (Phone) Fluorometric Measurements. Talanta.

[55] Loftsson T., Saokham P., Sá
Couto A. R. (2019). Self-Association of Cyclodextrins
and Cyclodextrin Complexes in Aqueous Solutions. Int. J. Pharm..

[56] Ramalho M. J., Andrade S., Coelho M. Á. N., Loureiro J. A., Pereira M. C. (2019). Biophysical
Interaction of Temozolomide and Its Active Metabolite with Biomembrane
Models: The Relevance of Drug-Membrane Interaction for Glioblastoma
Multiforme Therapy. Eur. J. Pharm. Biopharm..

[57] Stella V. J., Rao V. M., Zannou E. A., Zia V. (1999). Mechanisms of Drug
Release from Cyclodextrin Complexes. Adv. Drug
Delivery Rev..

[58] Waters L. J., Bedford S., Parkes G. M. B., Mitchell J. C. (2010). Influence of Lipophilicity
on Drug-Cyclodextrin Interactions: A Calorimetric Study. Thermochim. Acta.

[59] Lee S. Y. (2016). Temozolomide
Resistance in Glioblastoma Multiforme. Genes
Dis..

[60] Alonso M. M., Gomez-Manzano C., Bekele B. N., Yung W. K. A., Fueyo J. (2007). Adenovirus-Based
Strategies Overcome Temozolomide Resistance by Silencing the O6-Methylguanine-DNA
Methyltransferase Promoter. Cancer Res..

[61] Reoxcel. Reoxcel Hemostat; https://reoxcel.com/en/hemostat/reoxcel/.

[62] Yücel Medikal. Surgical Local Hemostats; https://yucelmedikal.com/en/clinisponge/.

[63] Corza Medical. Product Information Tachosil® medicated sponge – Singapore; https://corza.com/global/resources/product-information-tachosil-medicated-sponge-singapore/.

[64] Gheorghiţă D., Moldovan H., Robu A., Biţa A. I., Grosu E., Antoniac A., Corneschi I., Antoniac I., Bodog A. D., Băcilă C. I. (2023). Chitosan-Based
Biomaterials for Hemostatic Applications: A Review of Recent Advances. Int. J. Mol. Sci..

[65] Chen K.-Y., Chen Y.-C., Lin T.-H., Yang C.-Y., Kuo Y.-W., Lei U. (2020). Hemostatic Enhancement via Chitosan Is Independent of Classical Clotting
Pathways—a Quantitative Study. Polymers.

[66] Chen K. Y., Lin T. H., Yang C. Y., Kuo Y. W., Lei U. (2018). Mechanics
for the Adhesion and Aggregation of Red Blood Cells on Chitosan. J. Mech..

[67] Chou T. C., Fu E., Wu C. J., Yeh J. H. (2003). Chitosan Enhances Platelet Adhesion
and Aggregation. Biochem. Biophys. Res. Commun..

[68] Cines D. B., Lebedeva T., Nagaswami C., Hayes V., Massefski W., Litvinov R. I., Rauova L., Lowery T. J., Weisel J. W. (2014). Clot Contraction:
Compression of Erythrocytes into Tightly Packed Polyhedra and Redistribution
of Platelets and Fibrin. Blood.

[69] Tutwiler V., Mukhitov A. R., Peshkova A. D., Le Minh G., Khismatullin R. R., Vicksman J., Nagaswami C., Litvinov R. I., Weisel J. W. (2018). Shape Changes
of Erythrocytes during Blood Clot Contraction and the Structure of
Polyhedrocytes. Sci. Rep..

