# Supplementary material for: Multifunctional Hydrogel Flakes: An Innovative Approach to Localized Delivery of Temozolomide
Source: ACS Appl Mater Interfaces. 2025 Sep 9;17(38):53204–21. doi: 10.1021/acsami.5c13013 (PMC12464910; doi:10.1021/acsami.5c13013)
Supplement: Supplementary file 3 [file am5c13013_si_003.pdf]

Supporting Information

Multifunctional Hydrogel Flakes: An Innovative Approach to Localized Delivery of Temozolomide

Aleksandra Krajcer[1,2]*, Alicja Hinz[3], Monika Bzowska[3], Adrian Grzonka[2], Sylwia Stankiewicz[1,3], Kamil Kornaus[4], Bartosz Trzewik[2], Kinga Wójcik[5], Ewelina Grzywna[6], and Joanna Lewandowska-Łańcucka[2]*

[1] Doctoral School of Exact and Natural Sciences, Jagiellonian University, Prof. St. Łojasiewicza 11, 30-348 Kraków, Poland

[2] Faculty of Chemistry, Jagiellonian University, Gronostajowa 2, 30-387 Kraków, Poland

[3]Department of Cell Biochemistry, Faculty of Biochemistry, Biophysics and Biotechnology Jagiellonian University, Gronostajowa 7, 30-387 Kraków, Poland

[4] Faculty of Materials Science and Ceramics, AGH University of Krakow, Mickiewicza 30, 30-059 Kraków, Poland

[5]Faculty of Biochemistry, Biophysics and Biotechnology, Jagiellonian University, Gronostajowa 7, Kraków, Poland

[6]Department of Neurosurgery and Neurotraumatology, Jagiellonian University Medical College, Św. Anny 12, 31-008 Kraków, Poland

corresponding authors:
aleksandra.krajcer@doctoral.uj.edu.pl
lewandow@chemia.uj.edu.pl

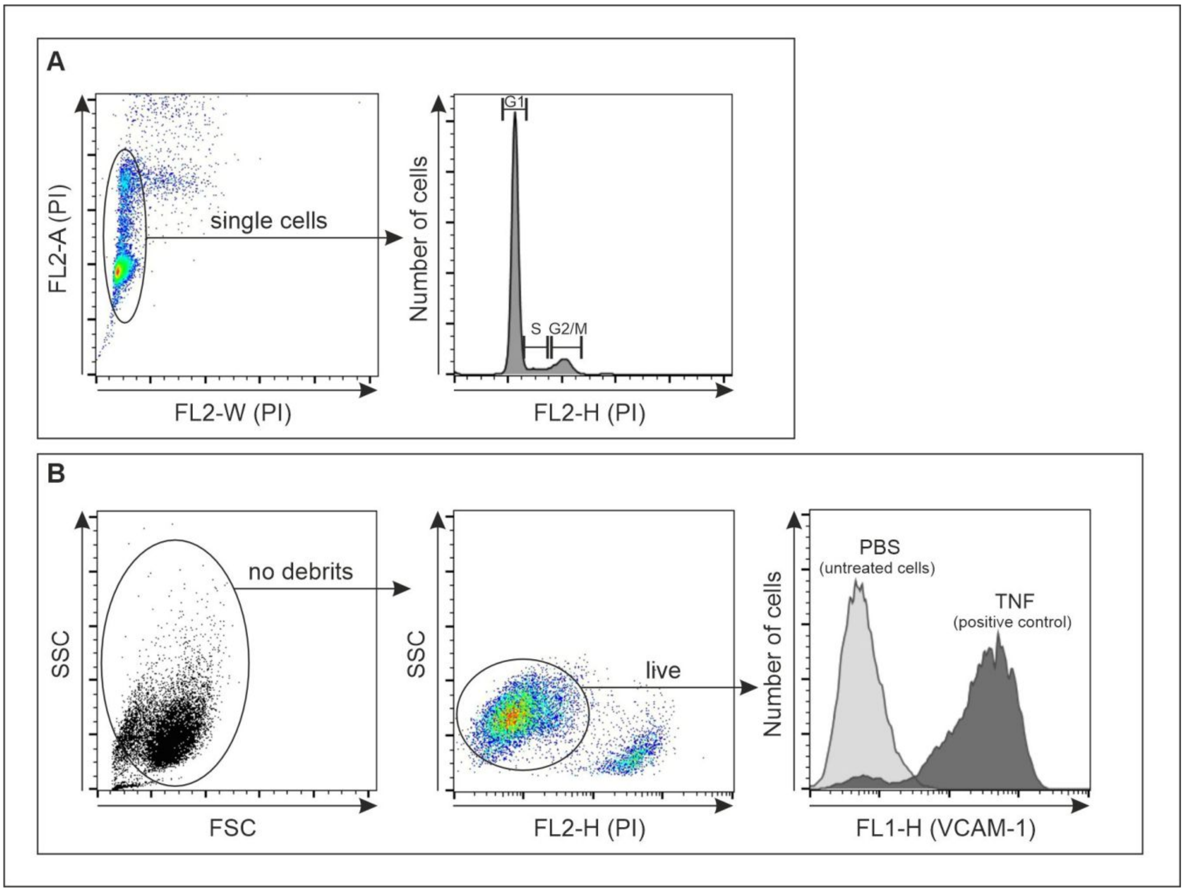

Figure S1. Gating strategy. (A) cell cycle analysis and (B) VCAM-1 measurement.

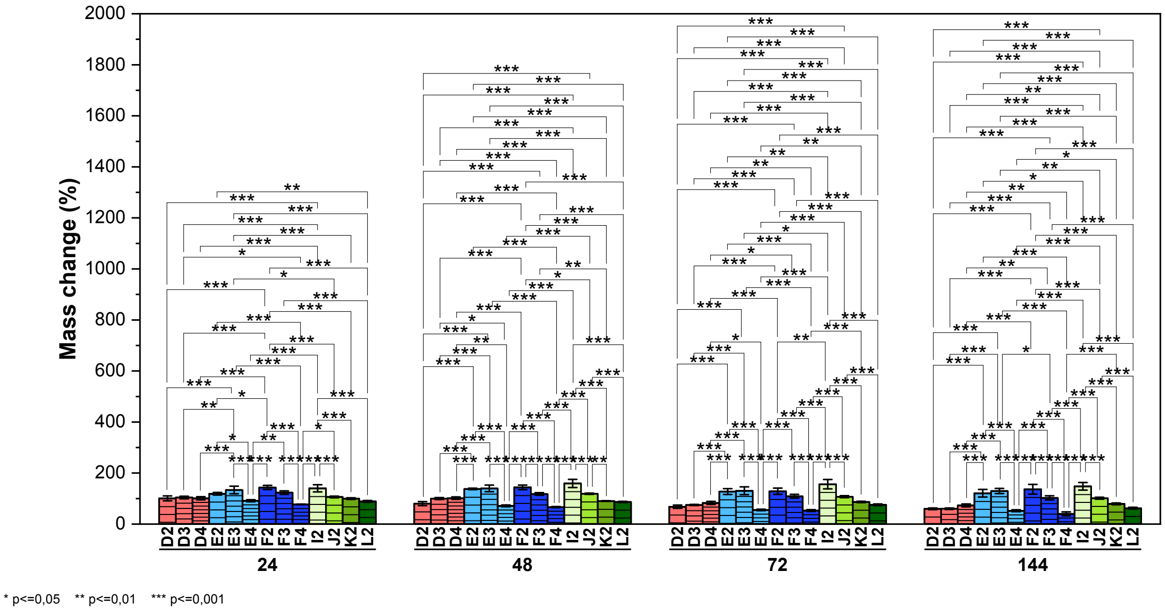

Figure S2. Degradation study results with statistical analysis using the Student's t-test.

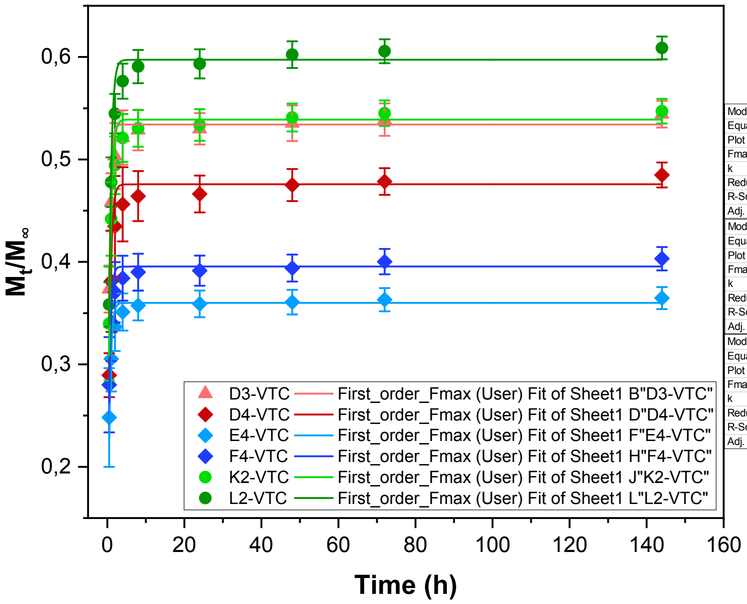

Model	First_order_Fmax (User)	Model	First_order_Fmax (User)
Equation	Fmax*(1 - exp(-k*x))	Equation	Fmax*(1 - exp(-k*x))
Plot	D3-VTC	Plot	D4-VTC
Fmax	0,53406 ± 0,00394	Fmax	0,47572 ± 0,00348
k	2,27635 ± 0,14612	k	1,83044 ± 0,11351
Reduced Chi-Sqr	0,34886	Reduced Chi-Sqr	0,27281
R-Square (COD)	0,95324	R-Square (COD)	0,97395
Adj. R-Square	0,94656	Adj. R-Square	0,97022
Model	First_order_Fmax (User)	Model	First_order_Fmax (User)
Equation	Fmax*(1 - exp(-k*x))	Equation	Fmax*(1 - exp(-k*x))
Plot	E4-VTC	Plot	F4-VTC
Fmax	0,35996 ± 0,00216	Fmax	0,39547 ± 0,00283
k	1,97595 ± 0,19916	k	2,18338 ± 0,26947
Reduced Chi-Sqr	0,17849	Reduced Chi-Sqr	0,24606
R-Square (COD)	0,86809	R-Square (COD)	0,82214
Adj. R-Square	0,84924	Adj. R-Square	0,79673
Model	First_order_Fmax (User)	Model	First_order_Fmax (User)
Equation	Fmax*(1 - exp(-k*x))	Equation	Fmax*(1 - exp(-k*x))
Plot	K2-VTC	Plot	L2-VTC
Fmax	0,53896 ± 0,00379	Fmax	0,59729 ± 0,00535
k	1,69936 ± 0,17797	k	1,67754 ± 0,13318
Reduced Chi-Sqr	0,40632	Reduced Chi-Sqr	0,9938
R-Square (COD)	0,87874	R-Square (COD)	0,95057
Adj. R-Square	0,86142	Adj. R-Square	0,94351

Figure S3. First-order model fit.

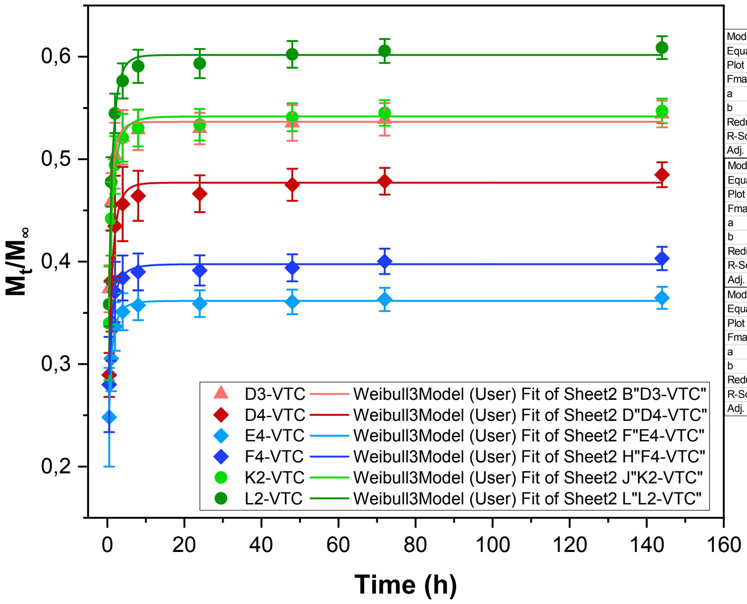

Model	Weibull3Model (User)	Model	Weibull3Model (User)
Equation	Fmax*(1 - exp(-(x^b / a)))	Equation	Fmax*(1 - exp(-(x^b / a)))
Plot	D3-VTC	Plot	D4-VTC
Fmax	0,53644 ± 0,00265	Fmax	0,47709 ± 0,00302
a	0,54467 ± 0,02671	a	0,6825 ± 0,04751
b	0,60455 ± 0,07557	b	0,6336 ± 0,09872
Reduced Chi-Sqr	0,13984	Reduced Chi-Sqr	0,1907
R-Square (COD)	0,98393	R-Square (COD)	0,98439
Adj. R-Square	0,97858	Adj. R-Square	0,97919
Model	Weibull3Model (User)	Model	Weibull3Model (User)
Equation	Fmax*(1 - exp(-(x^b / a)))	Equation	Fmax*(1 - exp(-(x^b / a)))
Plot	K2-VTC	Plot	L2-VTC
Fmax	0,5417 ± 0,0026	Fmax	0,6017 ± 0,00333
a	0,62601 ± 0,03503	a	0,67942 ± 0,02795
b	0,54933 ± 0,07859	b	0,64848 ± 0,06027
Reduced Chi-Sqr	0,16412	Reduced Chi-Sqr	0,32652
R-Square (COD)	0,95802	R-Square (COD)	0,98608
Adj. R-Square	0,94403	Adj. R-Square	0,98144
Model	Weibull3Model (User)	Model	Weibull3Model (User)
Equation	Fmax*(1 - exp(-(x^b / a)))	Equation	Fmax*(1 - exp(-(x^b / a)))
Plot	E4-VTC	Plot	F4-VTC
Fmax	0,36171 ± 0,00112	Fmax	0,39758 ± 0,00216
a	0,56045 ± 0,0218	a	0,54898 ± 0,03694
b	0,53406 ± 0,05584	b	0,47835 ± 0,08857
Reduced Chi-Sqr	0,04016	Reduced Chi-Sqr	0,11895
R-Square (COD)	0,97456	R-Square (COD)	0,9263
Adj. R-Square	0,96608	Adj. R-Square	0,90173

Figure S4. Weibull 3 model fit.

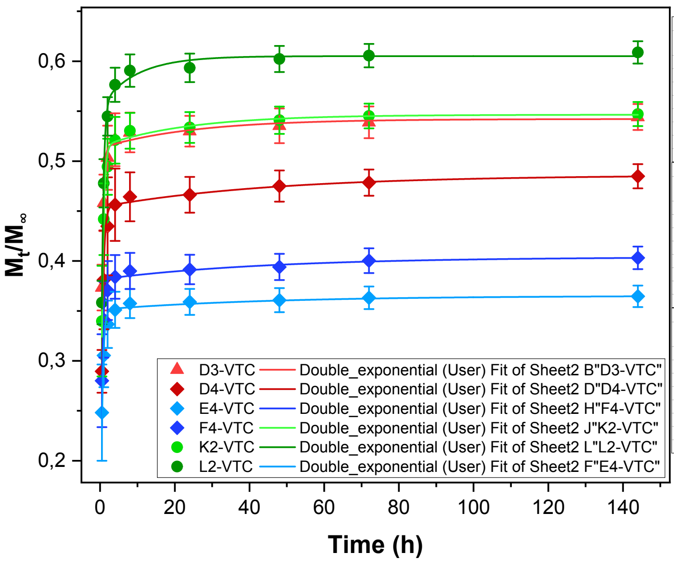

Model	Double_exponential (User)	Model	Double_exponential (User)
Equation	A1*(1 - exp(-k1*x)) + A2*(1 - exp(-k 2*x))	Equation	A1*(1 - exp(-k1*x)) + A2*(1 - exp(-k 2*x))
Plot	D3-VTC	Plot	D4-VTC
A1	0,51291 ± 0,00798	A1	0,45393 ± 0,00461
k1	2,51638 ± 0,13669	k1	2,0006 ± 0,06005
A2	0,02924 ± 0,008	A2	0,03214 ± 0,00466
k2	0,04028 ± 0,02704	k2	0,02144 ± 0,00825
Reduced Chi-Sqr	0,12622	Reduced Chi-Sqr	0,03666
R-Square (COD)	0,98792	R-Square (COD)	0,9975
Adj. R-Square	0,98067	Adj. R-Square	0,996

Model	Double_exponential (User)	Model	Double_exponential (User)
Equation	A1*(1 - exp(-k1*x)) + A2*(1 - exp(-k 2*x))	Equation	A1*(1 - exp(-k1*x)) + A2*(1 - exp(-k 2*x))
Plot	E4-VTC	Plot	F4-VTC
A1	0,3513 ± 0,0042	A1	0,38151 ± 0,00404
k1	2,16391 ± 0,18452	k1	2,46579 ± 0,18291
A2	0,01388 ± 0,00504	A2	0,0225 ± 0,00462
k2	0,02214 ± 0,02287	k2	0,02207 ± 0,01263
Reduced Chi-Sqr	0,08888	Reduced Chi-Sqr	0,05937
R-Square (COD)	0,95308	R-Square (COD)	0,96935
Adj. R-Square	0,92493	Adj. R-Square	0,95096

Model	Double_exponential (User)	Model	Double_exponential (User)
Equation	A1*(1 - exp(-k1*x)) + A2*(1 - exp(-k 2*x))	Equation	A1*(1 - exp(-k1*x)) + A2*(1 - exp(-k 2*x))
Plot	K2-VTC	Plot	L2-VTC
A1	0,51446 ± 0,00691	A1	0,55463 ± 0,01508
k1	1,9818 ± 0,13537	k1	1,99157 ± 0,12929
A2	0,03198 ± 0,00665	A2	0,05048 ± 0,01466
k2	0,04335 ± 0,02209	k2	0,10486 ± 0,06145
Reduced Chi-Sqr	0,09223	Reduced Chi-Sqr	0,21871
R-Square (COD)	0,98034	R-Square (COD)	0,99223
Adj. R-Square	0,96854	Adj. R-Square	0,98757

Figure S5. Double exponential model fit.

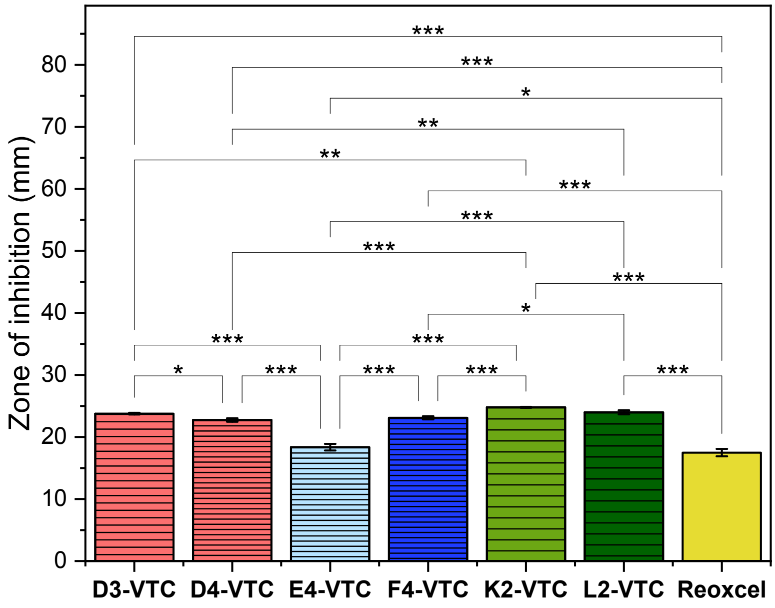

* p<=0,05 ** p<=0,01 *** p<=0,001

Figure S6. Antibacterial activity against Staphylococcus aureus, with statistical evaluation performed using Student's t-test.

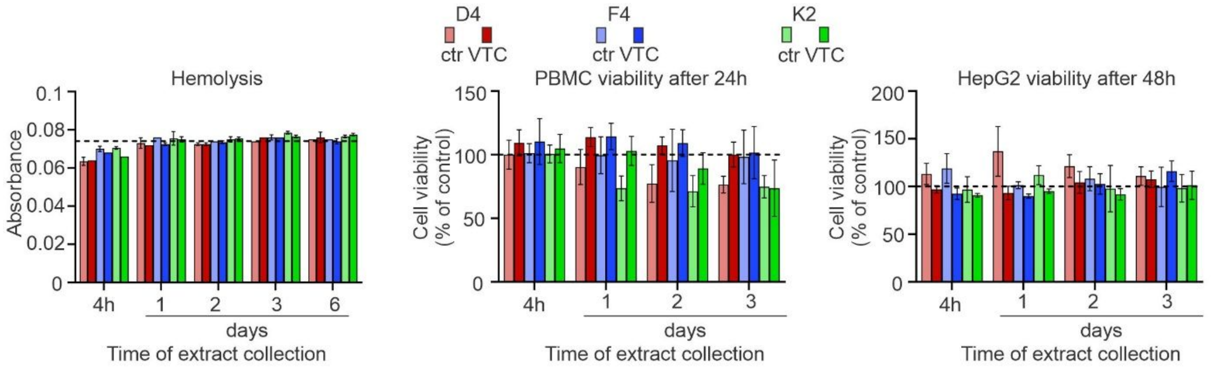

Figure S1. Biocompatibility analysis using extracts collected from materials at various time points and containing the potentially toxic compounds released from the materials. The hemolytic activity of materials verified using freshly isolated human red cells (the absorbance of 0.1% Triton X-100, representing total RBC hemolysis, was 0.44). Analysis of PBMC and HepG2 viability exposed to specific extract for 24h.

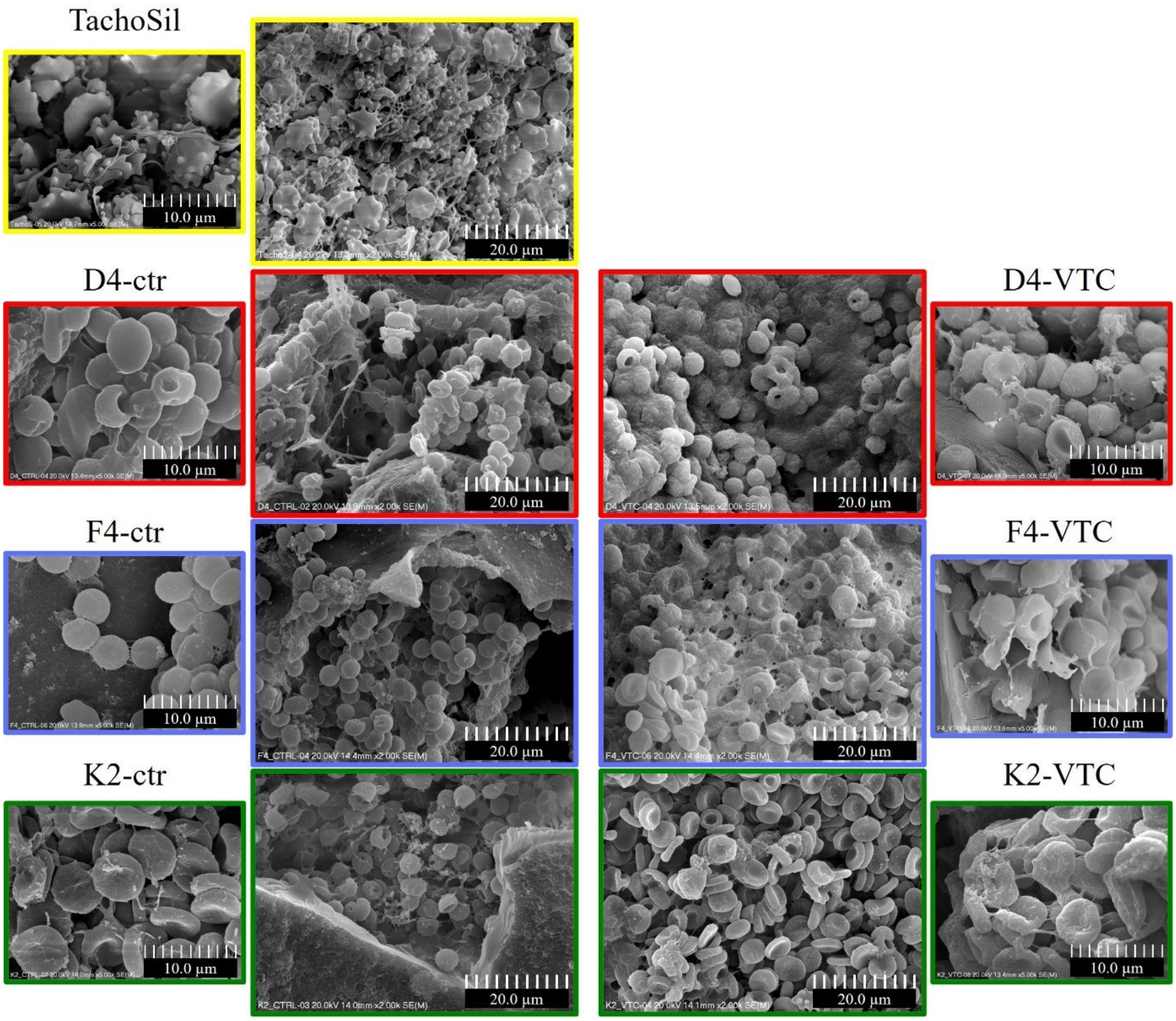

Figure S2. SEM images demonstrating clot formation inside the tested materials.

Movie S1. The movie presenting the developed materials’ blood absorption.

Movie S2. The movie presenting the commercial materials' blood absorption.
